# Examining the Impact of Digital Human Gaze Expressions on Engagement Induction

**DOI:** 10.3390/biomimetics8080610

**Published:** 2023-12-14

**Authors:** Subin Mok, Sung Park, Mincheol Whang

**Affiliations:** 1 Department of Emotion Engineering, Sangmyung University, Seoul 03016, Republic of Korea; ycd0611@gmail.com (S.M.); sjpark@smu.ac.kr (S.P.); 2 Department of Human-Centered Artificial Intelligence, Sangmyung University, Seoul 03016, Republic of Korea

**Keywords:** digital human, virtual human, avatar, ECA, gaze, eye contact, eye tracking, EEG, metaverse, engagement

## Abstract

With advancements in technology, digital humans are becoming increasingly sophisticated, with their application scope widening to include interactions with real people. However, research on expressions that facilitate natural engagement in interactions between real people and digital humans is scarce. With this study, we aimed to examine the differences in user engagement as measured by subjective evaluations, eye tracking, and electroencephalogram (EEG) responses relative to different gaze expressions in various conversational contexts. Conversational situations were categorized as face-to-face, face-to-video, and digital human interactions, with gaze expressions segmented into eye contact and gaze avoidance. Story stimuli incorporating twelve sentences verified to elicit positive and negative emotional responses were employed in the experiments after validation. A total of 45 participants (31 females and 14 males) underwent stimulation through positive and negative stories while exhibiting eye contact or gaze avoidance under each of the three conversational conditions. Engagement was assessed using subjective evaluation metrics in conjunction with measures of the subjects’ gaze and brainwave activity. The findings revealed engagement disparities between the face-to-face and digital-human conversation conditions. Notably, only positive stimuli elicited variations in engagement based on gaze expression across different conversation conditions. Gaze analysis corroborated the engagement differences, aligning with prior research on social sensitivity, but only in response to positive stimuli. This research departs from traditional studies of un-natural interactions with digital humans, focusing instead on interactions with digital humans designed to mimic the appearance of real humans. This study demonstrates the potential for gaze expression to induce engagement, regardless of the human or digital nature of the conversational dyads.

## 1. Introduction

As technology advances, the scope of applications for digital humans is rapidly expanding. Attempts to facilitate interactions between digital humans and people are not new. Beyond the realm of entertainment [1,2], digital humans have been employed in diverse fields, including clinical settings [3,4] and education [5,6,7]. To enable natural interactions with people, responses and expressions akin to human-to-human interactions are required. These responses and expressions change according to context, and appropriate contextual responses can induce engagement in the counterpart.

Engagement is a concept intimately linked with relationship formation and communication [8]. It involves investing the effort necessary to understand thoughts, evokes motivation and interest, and leads to behaviors such as collaboration [9]. It is not only significant in interactions between humans but is also pivotal in interactions between humans and robots, as well as between humans and digital humans.

When humans interact with agents, contextually appropriate engagement responses (e.g., gestures) from the agent can provide a positive experience for the user, and the agent’s implicit engagement responses (e.g., eye contact and gaze avoidance) have been shown to affect user engagement [10,11]. Therefore, for humans to have positive experiences and engage in natural interactions and communication with digital humans, it is essential for digital humans to be capable of inducing engagement.

## 2. Related Works

### 2.1. Immersion and Engagement as a Social Construct

Digital humans serve as avatars, i.e., ‘digital stand-ins’ for the user [12] or virtual humans, which are defined as characters on a screen that bear human-like appearances [13,14,15]. These definitions correspond to the concept of digital humans, whose immersive presence can motivate learners in a virtual world or facilitate engagement with other digital entities, enhancing immersion in virtual environments [16,17]. Immersion is defined as a state of concentration in which one is enveloped in an environment that provides continuous stimuli and experience, accompanied by intense focus and a distorted sense of time [18,19].

The immersion between digital humans and people usually acts as a social immersion [20], where immersion occurs in the interaction, potentially including emotional elements and empathy [20,21,22]. Empathy in interaction encompasses both cognitive and emotional responses to other people [23,24,25,26].

While immersion is a more encompassing experience where people become fully absorbed in social interaction, often leading to a temporary loss of awareness of their physical surroundings, engagement involves active and emotional participation; that is, engagement can be viewed as a precursor of immersion.

In [9], engagement is conceptualized as comprising two main states: individual and social states. Johnston delineates social state engagement as a collective experience within a group characterized by elements like collective action; participant power; and cognitive–emotional forms, including orientation, intention, and experience. In contrast, individual state engagement consists of three dynamic dimensions: the cognitive, affective, and behavioral dimensions. The cognitive dimension involves a person’s effort in understanding complex ideas and skills, and their focus and interest in a subject. The affective dimension covers emotional responses, with valence engagement indicating attraction or aversion, thereby influencing motivation or interest. The behavioral dimension includes actions and collaboration and is shaped by cognitive or emotional engagement.

This conceptual framework intersects with ongoing discussions among researchers regarding the definition of engagement in contexts involving digital humans and people. The author of [27] defines user engagement as the quality of user experience marked by cognitive, emotional, and temporal investment when interacting with digital systems. Criteria for narrative engagement include narrative understanding, attentional focus, emotional engagement, and narrative presence [22]. Standards for video game engagement encompass focused attention, perceived usability, aesthetics, and satisfaction [28]. Moreover, nonverbal involvement engagement is assessed through scales like immediacy, expressiveness, altercentrism, interaction management, composure, and positive affect [29].

In this study, we integrate these diverse perspectives, identifying common threads in the definitions of engagement. We categorize these into immersion (covering cognitive, focused, understanding, and altercentrism aspects), empathic (including emotional empathic components), and emotional (emphasizing positive affective responses) dimensions. Our analysis focuses on aspects such as identification and cognitive empathy, as well as the broader emotional aspects of a conversation, with a particular emphasis on enjoyment and affective response.

### 2.2. Conversation with Digital Humans

In this research, the interaction scenarios between digital humans and individuals are specifically defined as conversational contexts. We aim to investigate the differences in engagement between human-to-human conversations and human-to-digital human dialogues. The conversational contexts are categorized as face-to-face human interactions, where individuals communicate directly with each other, and digital human interactions, where participants engage in dialogue with digital humans.

However, unlike face-to-face human conversations, where participants share the same physical space, interactions with digital humans are limited by current technology, preventing physical copresence. Therefore, instead of a direct face-to-face dialogue with another human, interactions with digital humans occur through a medium such as a monitor, which could influence the outcome. This has led to the exploration of mediated communication through monitors in non-face-to-face conversational scenarios. In these cases, non-face-to-face dialogue is defined as interactions in which a real person is represented on a monitor and converses with another person. 

Consequently, our study delineates three distinct conversational contexts: face-to-face conversations, where individuals directly engage with one another; non-face-to-face conversations, where individuals communicate via video; and digital-human conversations, where a person interacts with a digital human through video.

### 2.3. Gaze Expression of Digital Humans

Eye gaze is recognized as a critical component for the establishment of relationships with others, transmission of information, acquisition of new knowledge, and discernment of various intents and information related the actions of others [30,31]. Generally, gaze plays a key role in controlling interactions and smoothly coordinating social exchanges [32].

The role of gaze within the context of immersion extends beyond human-to-human interactions and can be applied to human-robot interactions. Studies have reported that the expression of immersive gestures such as eye contact in human–robot interactions has a positive impact compared to when such gestures are absent [21,33,34].

Research findings suggest that gaze expression is a crucial cue for coordination of interactions and sharing of one’s state with another party—not only in human-to-human interactions but also in interactions involving non-human entities like robots. Given the wide range of gaze expressions influenced by variables such as eye movement and speed, this study narrows the scope of gaze expressions to instances of eye contact and gaze avoidance in dyadic conversations.

### 2.4. Research Hypotheses

For digital humans to communicate naturally with people, it is necessary to induce a sense of engagement in the human user. Prior research has established that gaze expressions such as eye contact and avoidance can play a significant role in fostering engagement [11,21,33]. However, there is a paucity of comparative studies on how user engagement varies across face-to-face, non-face-to-face (i.e., face-to-video), and digital-human conversational contexts.

With this study, we aim to evaluate the differences in user engagement in face-to-face, face-to-video, and digital-human conversational scenarios, as well as the engagement effect of gaze expressions such as eye contact and avoidance, using subjective evaluation methods. We also intend to examine how implicit responses change by measuring gaze variables and brain waves.

Hypothesis H1 seeks to identify differences in engagement based on the conversational context when gaze expression (eye contact or gaze avoidance) is held constant. Subhypothesis H1-1 posits that there are differences in engagement according to the conversational context when eye contact is expressed. H1-2 hypothesizes that there are differences in engagement according to the conversational context when gaze avoidance is expressed.

**Figure 1:**
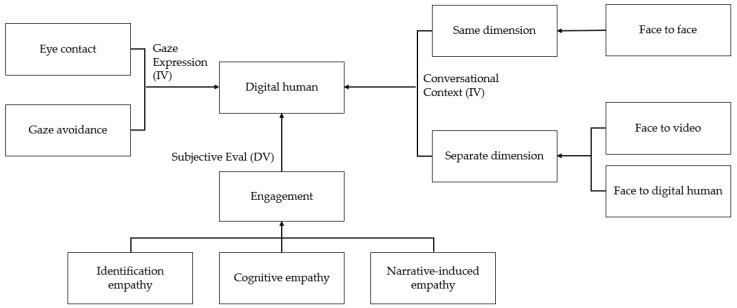
Modal diagram of the investigation.

Hypothesis H2 seeks to identify differences in engagement based on gaze expressions (eye contact and gaze avoidance) when the conversational context is held constant. Subhypothesis H2-1 posits that there are differences in engagement according to gaze expressions in face-to-face conversations. H2-2 hypothesizes that there are differences in engagement according to gaze expressions in face-to-video conversations. H2-3 hypothesizes that there are differences in engagement according to gaze expressions in digital-human conversations. Lastly, this research explores how engagement may vary with the emotional content of the stimuli; that is, H3 hypothesizes that there are differences in engagement depending on positive and negative emotional content. Figure 1 presents the structural organization of the variables outlined in the hypotheses. Details regarding the subjective evaluations are elaborated in Section 3.3, ‘Subjective Measurements’.

## 3. Materials and Methods

### 3.1. Participants

In this study, two independent variables were examined (see Figure 1). The first pertains to gaze expressions, consisting of two levels: eye contact and eye avoidance. The second independent variable pertains to the type of interaction and is categorized into three levels: face-to-face conversation, face-to-video conversation, and face-to-digital human conversation. The first two conditions involved a conversation with a human (i.e., experimenter), and the latter involved a conversation with a digital human. Using power analysis program MorePower, the power set of the repeated-measures ANOVA was analyzed (with power at 0.8, α = 0.05, and a Cohen’s d effect size of 0.4). The results indicate that a sample size of approximately 34 is necessary to achieve adequate statistical power. To ensure robustness, we recruited 45 university students. The age of the participants ranged from 20 to 30 years (mean age = 23.56; SD = 2.99). The sample comprised 14 men and 31 women. Participants with a corrective vision ≥0.7 were chosen, and those with visual deficiencies were excluded to ensure consistent recognition of visual stimuli.

We advised participants to get sufficient sleep and to abstain from smoking, as well as alcohol and caffeine intake, on the day before the experiment. To ensure accurate detection of gaze movements, we placed restrictions on the wearing of glasses and the application of heavy makeup. All participants were informed about the experiment’s objectives and procedures. After gaining a comprehensive understanding, they provided signed consent. Participants were compensated for their time and contribution by payment of a fee.

### 3.2. Stimuli

#### 3.2.1. Video Stimuli

Both the face-to-video and face-to-digital human conversations utilized a prerecorded video stimulus. The face-to-video stimulus was recorded with the human experimenter using an iPhone 12 Pro. It was crucial for the experimenter in the video to appear as if she was making eye contact. To simulate eye contact, a photo of the experimenter, printed in actual face size was used. A camera was strategically positioned by creating a hole between the eyes in the photo. The experimenter then recorded the video while focusing her gaze on the eyes in the photograph.

Drawing from gaze-avoidance research [35,36], the gaze direction was shifted either to the left or the right to demonstrate gaze avoidance. For gaze-avoidance representation, it is emphasized that the gaze remains steady in one direction. This is because the intended effect might be compromised if the gaze shifts, such as by looking in another direction without fixating. Throughout the filming of the experimental stimulus, the experimenter ensured consistency in hair styling, makeup, clothing, camera positioning, and posture.

#### 3.2.2. Digital Human Design

The digital human used in the experiment was modeled using Character Creator 3 (Reallusion, San Jose, CA, USA, 2018), Headshot Plug-in (Reallusion, 2019), and Hair Builder (Reallusion, 2021). The digital human’s facial features and hair were designed to match the shape of those of the real human featured in the experiment’s video stimulus (i.e., face-to-video conversation).

**Figure 2:**
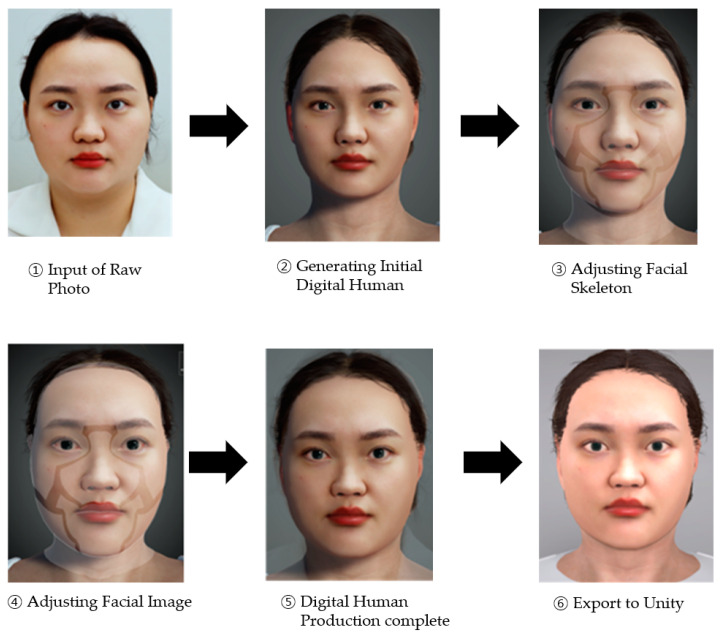
The process of creating the digital human.

Figure 2 illustrates the process of generating a digital human using ‘Character Creator 3’ software. This process began with importing a photograph of the experimenter’s face into the program. Subsequent adjustments were made to the facial skeleton and hair to closely align the digital human with the physical characteristics of the experimenter’s photograph. Specific modifications included the eyes, pupils, ears, lips, and nose to ensure a high degree of resemblance. In this study, we aimed to replicate the experimenter’s appearance and hairstyle accurately within the digital human creation. The finalized digital human was then exported from Character Creator 3 (CC3) for integration into the Unity environment.

**Figure 3:**
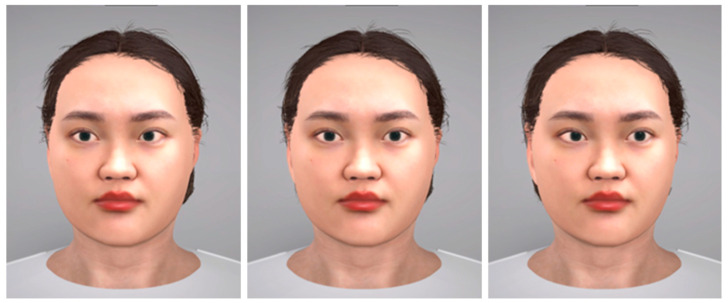
Directions of the digital human’s eyes: (**left**) gaze avoidance with eyes directed towards the left; (**middle**) making eye contact; (**right**) gaze avoidance with eyes directed towards the right.

Figure 3 illustrates the methodology employed in the design and development of nonverbal expressions, specifically gaze avoidance and eye contact, for the digital human construct. We replicated the natural tendency of the experimenter to couple head orientation with gaze direction during gaze-avoidance behavior. We calibrated the gaze dynamics of the virtual entity to incorporate these concurrent head movements, enhancing the realism of the simulation.

The animation of the digital human’s head and gaze movements was executed using Unity. In parallel, the lip synchronization of the digital human was also engineered within Unity, ensuring that all facial animations were coherent with the spoken dialogue. This lip-sync process was tailored to emulate the experimenter’s distinct speaking patterns, informed by analysis of face-to-video stimulus. To achieve a comprehensive integration of these elements, animated head and gaze movements, alongside lip syncing, were synchronized and manipulated through Unity, then rendered as visual stimuli.

#### 3.2.3. Sentence Stimuli

In this study, we utilized verbal stimuli derived from [37], comprising English sentences designed to evoke positive and negative emotions. These sentences were translated from English to Korean and subsequently elaborated upon to extend their spoken duration to 30 s through the addition of further detail. The authenticity of the emotional conveyance in the Korean translations was validated by 31 participants using a 9-point Likert scale as a manipulation check. Statistical analysis confirmed that the Korean sentences effectively captured the intended emotional valence, be it positive or negative (t = 28.410, *p* < 0.001). Table 1 and Table 2 present the selection of the top-six sentences for the positive and negative categories, respectively, that achieved the highest scores for their emotional content according to the Likert-scale results.

### 3.3. Subjective Measurements

This study was designed to explore conversational engagement between two conversational dyads. Prior to this work, the literature on the development of specific engagement measurement tools has been limited, with notable exceptions found in areas such as user experience [27], gaming user experience [28], student engagement [36], story engagement [22], and broader user experience engagement metrics.

In our investigation, we gathered sentences from studies on user experience interactions [27,36] and game user experience [28]. These sentences were translated into Korean and rephrased to align with our conceptualization of engagement. Furthermore, we incorporated empathy scales to quantify empathetic responses, as our definition of engagement includes an empathy component [38]. Identification empathy refers to a deep emotional connection through which an individual feels as if they are personally experiencing the emotions and experiences of another person [39,40]. On the other hand, cognitive empathy involves understanding and adopting another person’s perspective, enabling one to infer their emotions, thoughts, and personality traits [24,41,42].

A cohort of 31 participants was enlisted to assess engagement levels. Participants were exposed to stimulus videos containing either positive or negative narratives and subsequently rated their engagement using a designated scale. The study proceeded with a validation phase through factor and reliability analysis.

The results, as depicted in Table 3, indicate a segmentation into four distinct factors through factor analysis: engagement, identification empathy, cognitive empathy, and narrative-induced emotion. The Cronbach’s α metric was utilized to evaluate reliability, with a value of 0.6 or above generally accepted as indicative of sound reliability.

Our findings revealed a Cronbach’s α value for concentration of 0.956, emotional empathy of 0.905, cognitive empathy of 0.787, and narrative emotion of 0.912. These results affirm that each construct exhibited a reliability of 0.6 or greater, thereby substantiating the robustness of our measures.

### 3.4. Experimental Procedure

We created the digital human using a software program (Character Creator 3, Reallusion Inc., 2018; Headshot Plug-in, Reallusion Inc., 2019) and manipulated video stimuli using a program written in C# (Unity, Unity Technologies, Austin, TX, USA, 2021). The stimuli were displayed on a 27-inch LCD monitor, except for the face-to-face conversation. For the face-to-face conversation, the a monitor of the same size was utilized to track participants’ eye movements.

In both face-to-video and face-to-digital human conversations, participants used an adjustable desk to align their gaze with that of the photo stimuli until eye contact was established. This method was informed by the authors of [36], who allowed participants to adjust the desk height to achieve eye contact.

Electroencephalogram (EEG) measurements were taken using an instrument (Model 202, Mitsar Inc., St. Petersburg, Russia) configured to the 10–20 system with eighteen channels (FP1, FP2, F3, F4, Fz, F7, F8, T3, T4, C3, C4, T5, T6, Pz, P3, P4, O1, and O2), recording EEG signals at a sampling rate of 500Hz with reference to the Cz electrode. In addition, we tracked gaze and eye movement at 60Hz using equipment from GazePoint (Model GP3, Canada). A motion desk was also employed to facilitate the participants’ gaze adjustment for eye contact with the stimuli. This setup procedure was completed prior to exposure to the stimuli.

**Figure 4:**
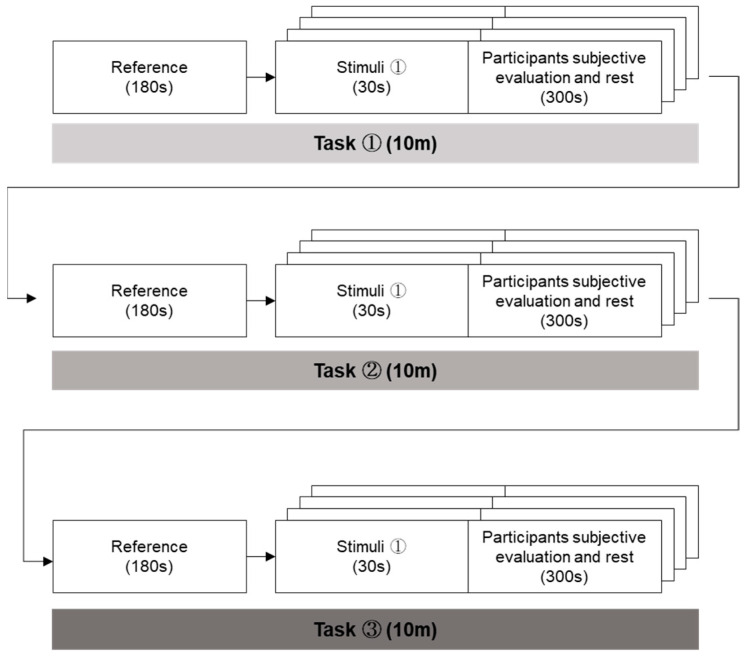
Experimental procedure.

Figure 4 provides a comprehensive overview of the experimental procedure. We implemented the protocol outlined in the figure to allow a participant to undergo exposure to face-to-face, face-to-video, and face-to-digital conversations within a controlled experimental setting. Before the onset of stimulus exposure, we gathered baseline data for a duration of 180 s. The participants engaged in a total of 12 dialogues, which included an equal division (i.e., four) of four face-to-face, four face-to-video, and four face-to-digital human conversations. To mitigate any potential sequencing biases (i.e., ordering effects), the order of these stimuli was randomized across the conversations. Following the viewing of each stimulus, participants were given a 5 min intermission. In these intervals, we efficiently gathered the participants’ emotional responses and subjective feedback, employing a 7-point Likert scale.

**Figure 5:**
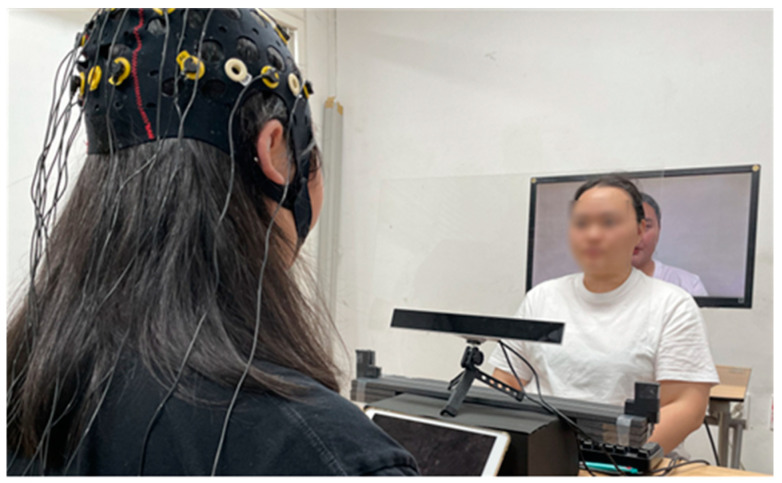
Setup for the face-to-face conversation.

Figure 5 illustrates the setup for the face-to-face conversation. The monitor used to measure the participants’ gaze was placed on a table one meter away from the participants. Additionally, the gaze tracker was positioned on the table at a distance of 40 cm from the participants. To ensure the collection of high-quality gaze data, the height of the gaze tracker was carefully adjusted and positioned directly below the monitor. An acrylic barrier was placed between the participant and the experimenter as a precautionary measure to prevent the spread of COVID-19.

**Figure 6:**
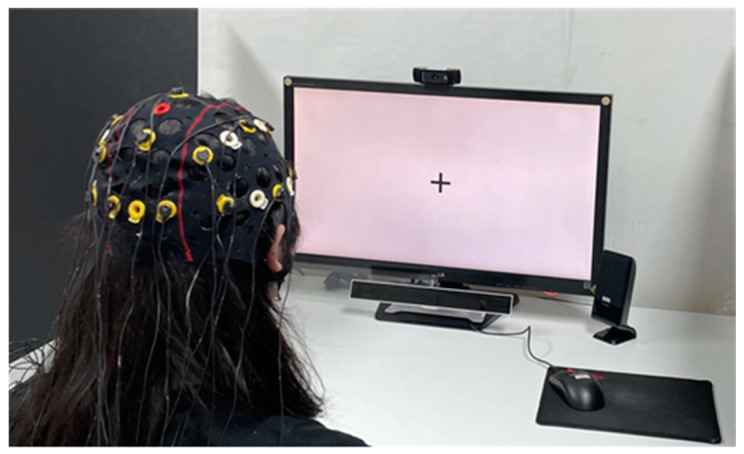
Setup for both face-to-video and face-to-digital human conversations.

Figure 6 depicts the setups for both face-to-video and face-to-digital human conversations. The monitor utilized to track participants’ gazes, as well as to display stimuli, was positioned on a table one meter away from the participants, with the gaze tracker placed beneath it. Data on the participants’ brain activity and eye movements were simultaneously captured using an EEG cap and the gaze tracker.

### 3.5. Data Analysis

In this study, we aimed to understand the differences in user immersion and emotion regarding conversational situations and gaze expressions. Since this study involved a 2-factor, repeated-measures, within-subject design, we conducted the analysis using a two-way repeated-measures ANOVA within-subject method.

We excluded data from 11 participants with poor eye-tracking quality during the data collection process. The criteria for poor eye-tracking quality were established as follows. We excluded participant data when, after calibration, the eye-tracking camera completely failed to capture gaze during video viewing or when the participants’ movements during video viewing were so pronounced that they compromised the calibration information.

To compare conversational situations and gaze expression methods and examine the interaction effects between the two factors, we performed statistical analysis using SPSS 21 (IBM, Armonk, NY, USA). When the data met the assumption of normality, we conducted a repeated-measures ANOVA, and when they did not, we proceeded with the non-parametric Friedman test.

#### 3.5.1. Brain Waves

EEG signals underwent processing through a band-pass filter (BPF) set to a range of 1–50 Hz. Subsequently, the EEG spectrum was examined utilizing the fast Fourier transform (FFT) technique. In this analysis, the EEG spectrum was categorized into distinct frequency bands, namely: delta (1–4 Hz), theta (4–8 Hz), alpha (8–13 Hz), and beta (13–20 Hz) [43,44]. The band power for each range was determined by aggregating the power values of frequencies from each spectral dataset. The relative power of each frequency band, ranging from delta to beta, was determined by calculating the ratio of the total power to the power in each specific band, as illustrated in Equation (1).

(1) $$each\;band\;ratio\left(\%\right)=\frac{each\;band\;power}{total\;power}\times 100(\%)$$

We were interested in any significant differences in response based on gaze expression and conversational situations regarding relative power across electrode locations and frequency bands.

#### 3.5.2. Gaze

**Figure 7:**
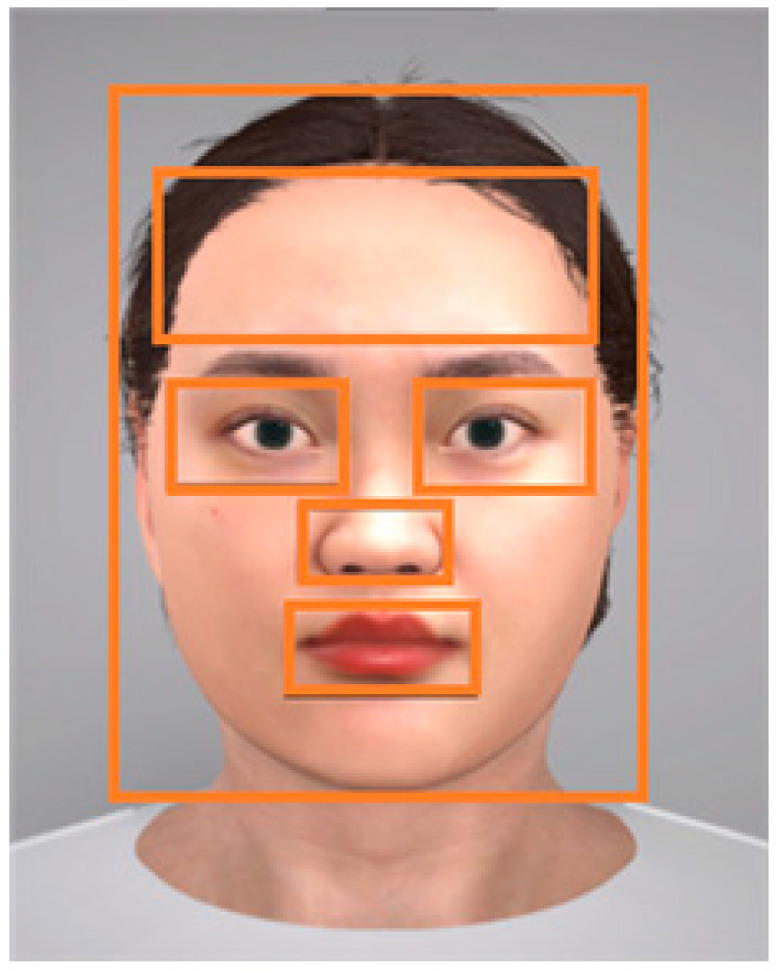
The segmentation of ROIs: left eye, right eye, nose, mouth, forehead, and overall face.

Figure 7 illustrates the segmentation of the region of interest (ROI) for the purpose of evaluating the gaze patterns of our participants. The delineated ROIs include the left eye, right eye, nose, mouth, forehead, and overall face. We assessed gaze variations through three distinct metrics: fixation count, revisit count, and the proportion of gaze time allocated to each ROI.

The fixation count refers to the frequency with which a participant’s gaze remained steady within a single ROI. The revisit count is the number of instances of a participant’s gaze returning to a previously focused ROI after momentarily shifting to a different area. The ratio of an ROI to the total gaze is the quotient of the fixation count on a given ROI divided by the sum of fixations across all ROIs, providing a normalized measure of attention allocation.

Our objective was to quantify these gaze metrics—fixation, revisit, and ROI ratio—to identify any variances in gaze patterns attributable to differing conversational contexts and gaze expressions among the participants.

## 4. Results

### 4.1. Subjective Evaluation Results

#### 4.1.1. Positive Stimuli

We conducted a subjective evaluation of participants’ responses to positive stimuli. Analysis of the interaction effect between conversational situations and gaze expressions indicated no significant difference in engagement (F(2, 99) = 2.806, *p* > 0.05).

**Figure 8:**
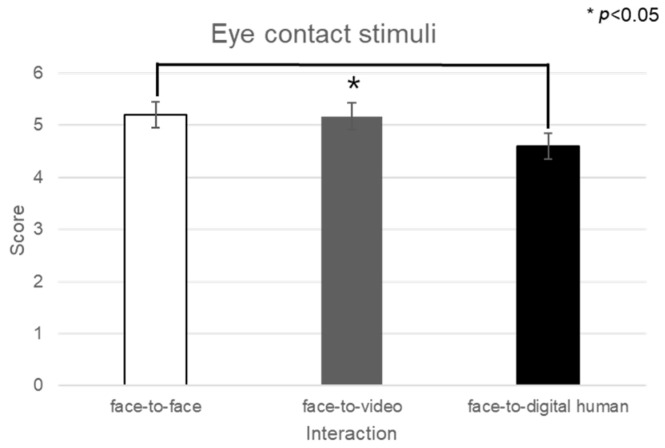
Analysis of subjective evaluation of participants’ responses to positive stimuli.

Upon examining the engagement levels across three types of conversational contexts—face-to-face, face-to-video, and face-to-digital human interactions—a significant main effect emerged (F(2, 99) = 3.851, *p* < 0.05), as illuminated in Figure 8. Subsequent post hoc comparisons highlighted that there was no significant difference in engagement between the face-to-face and face-to-digital human settings (*p* > 0.05).

**Figure 9:**
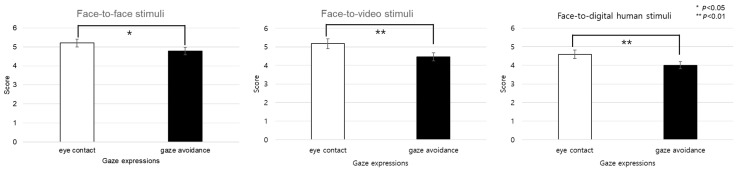
Analysis of subjective engagement scores between eye contact and gaze avoidance.

Figure 9 graphically represents the subjective engagement scores, delineating the impact of conversational scenarios and gaze expressions under conditions involving positive stimuli. Further analysis was conducted to assess participant responses to eye contact versus gaze-avoidance stimuli. The findings indicated that participants exhibited greater engagement with eye-contact stimuli as opposed to gaze-avoidance stimuli across all conversational situations: face-to-face (t(99) = 2.136, *p* < 0.05), face-to-video (t(99) = 3.740, *p* < 0.01), and face-to-digital human (t(99) = 3.600, *p* < 0.01).

#### 4.1.2. Negative Stimuli

**Figure 10:**
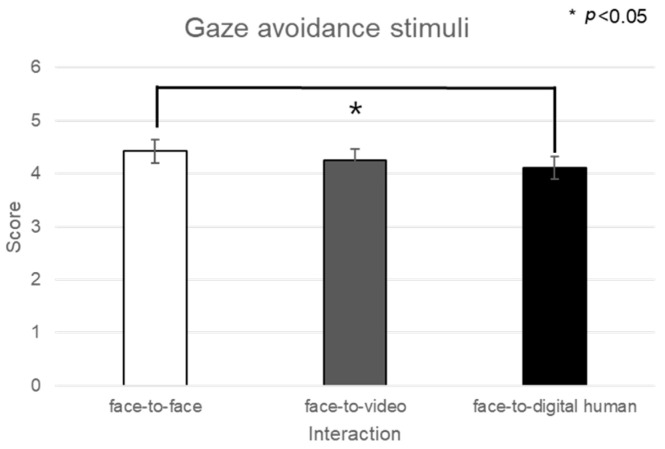
Analysis of subjective evaluation of participants’ responses to negative stimuli.

Figure 10 details the outcomes of the subjective engagement assessments, highlighting distinctions among conversational situations under conditions involving negative stimuli. The results indicate that the interaction between conversational contexts and gaze expressions did not yield a statistically significant difference in engagement levels (F(2, 99) = 1.992, *p* > 0.05). However, when examining the three different conversational situations—face-to-face, face-to-video, and face-to-digital human interactions—a significant main effect was detected (F(2, 99) = 3.637, *p* < 0.05).

Subsequent post hoc analysis identified a significant variation in engagement during the face-to-face and face-to-digital human conversational scenarios when negative stimuli were introduced (*p* < 0.05). Nonetheless, differences in engagement between gaze-oriented and gaze-avoidance expressions were not statistically significant.

### 4.2. Brainwave Results

We computed the relative power for each frequency band across 18 brainwave channels and conducted statistical analysis to examine brain responses to different stimuli. We found significant results specifically for channels fp1, f1, f7, t3, and t4. These channels were chosen for their relevance to the cognitive functions engaged during interaction with a virtual agent in a conversational setting. Channels fp1, f1, and f7 are associated with the frontal lobe, which encompasses a range of cognitive processes, including those imperative for the interactive tasks involved in our experiment. Particularly, fp1 involves the prefrontal cortex, which is known for its role in complex cognitive tasks and executive functions. Channels t3 and t4, which are related to the temporal lobe, are essential for semantic processing and language, both of which are fundamental elements of conversation. By concentrating our analysis on these channels, we were able to derive meaningful and hypothesis-driven insights into the neural basis of human–digital human interaction.

#### 4.2.1. Positive Stimuli

**Figure 11:**
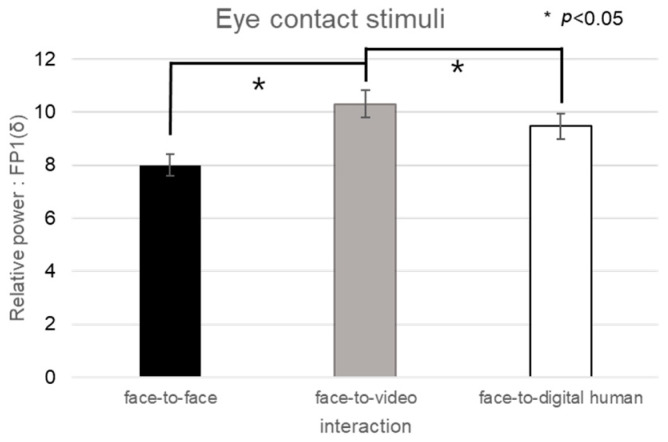

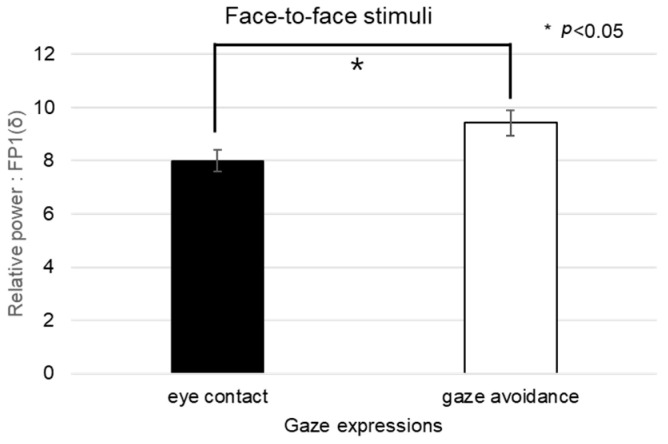
Analysis of brainwave channel FP1 in response to positive stimuli.

In the context of positive stimuli, the distribution of delta-band power in channel FP1 did not conform to normality in conversational scenarios; hence, nonparametric Friedman tests were utilized (see Figure 11).

In the analysis of eye contact versus gaze-avoidance stimuli, significant differences were detected in the face-to-face situation (z = −2.060, *p* < 0.05). Significant disparities in the delta-band power were noted when participants were exposed to eye-contact stimuli, specifically between face-to-face and face-to-video (z = −2.129, *p* < 0.05) and between face-to-video and face-to-digital human scenarios (z = −2.197, *p* < 0.05).

**Figure 12:**
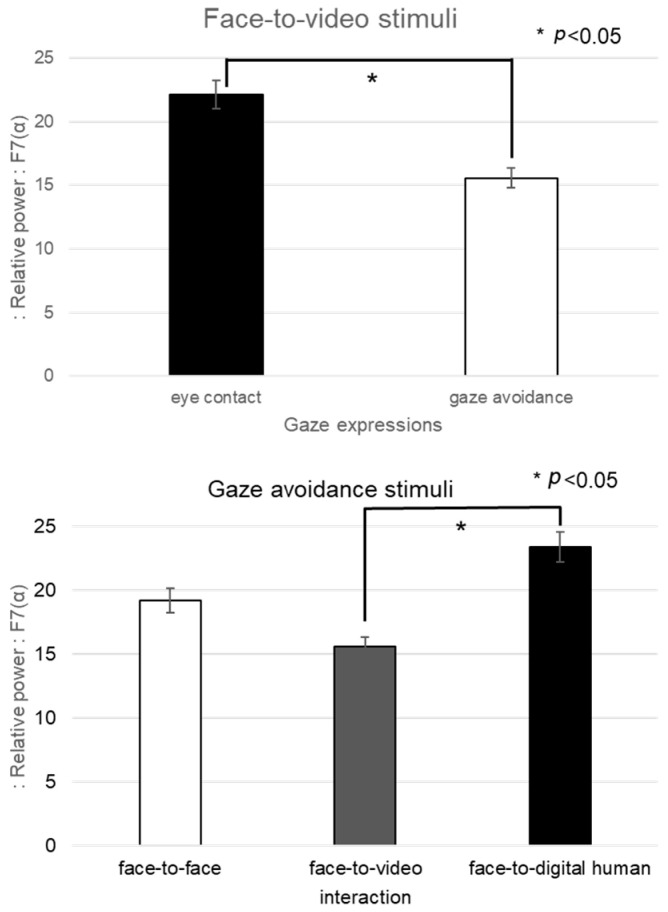
Analysis of brainwave channel F7 in response to positive stimuli.

For the alpha band in channel F7, given the nonparametric nature of the normality test results, a Friedman test was conducted, as outlined in Figure 12. The analysis indicated a significant main effect in the brain’s response to gaze-avoidance stimuli across the face-to-face, face-to-video, and face-to-digital human conversational scenarios (*p* < 0.05). Additional post hoc tests were administered for further examination.

The post hoc analysis pointed out notable differences between the face-to-video and face-to-digital human scenarios (z = −2.351, *p* < 0.05) in the context of gaze-avoidance stimuli. Moreover, a significant contrast was observed between responses to gaze-aligned and gaze-avoidance stimuli under the face-to-video condition (z = −2.282, *p* < 0.05).

#### 4.2.2. Negative Stimuli

**Figure 13:**
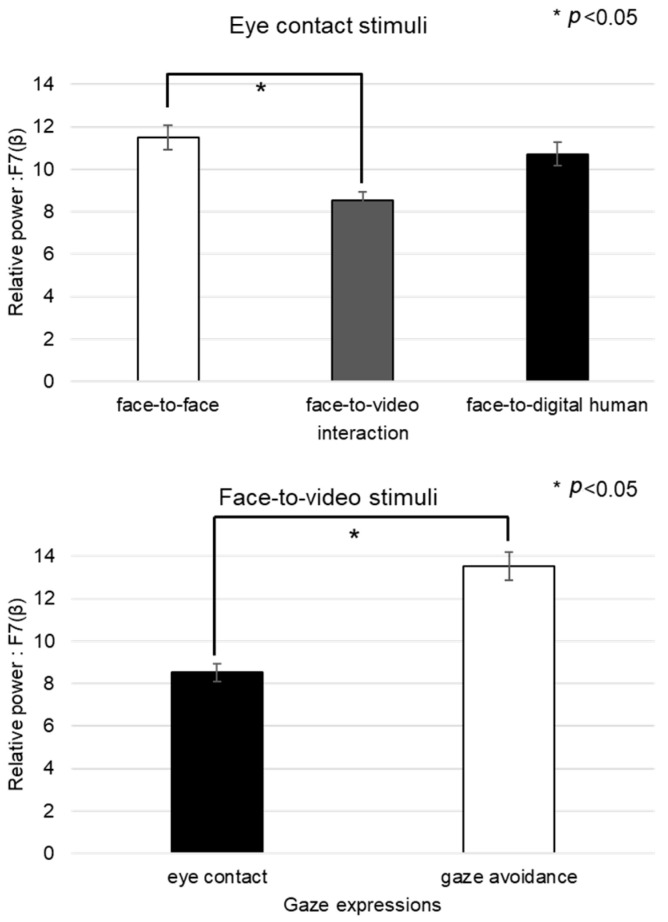
Analysis of brainwave channel F7 in response to negative stimuli.

For the beta-band data from channel F7 during exposure to negative stimuli, the normality test indicated a nonparametric distribution, leading us to employ the Friedman test for analysis (see Figure 13). Significant main effects were found when eye-contact stimuli were presented across three conversational situations: face-to-face, face-to-video, and face-to-digital human scenarios (*p* < 0.05). Subsequent post hoc analysis revealed significant differences specifically between the face-to-face and face-to-video conditions (z = −2.009, *p* < 0.05). Furthermore, within the face-to-video setting, significant variations were noted between responses to gaze alignment and gaze avoidance (z = −2.077, *p* < 0.05).

**Figure 14:**
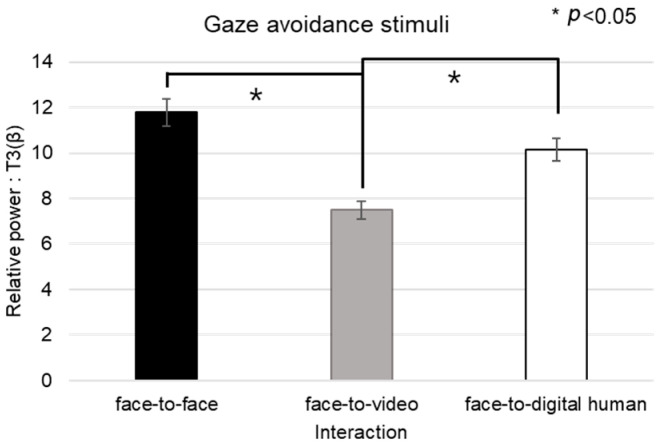

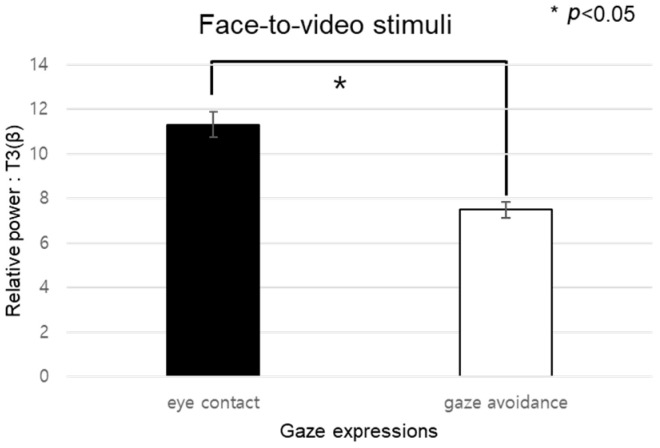
Analysis of brainwave channel T3 in response to negative stimuli.

Regarding the beta band in channel T3 under the conditions of gaze-avoidance stimuli, nonparametric distributions necessitated a Friedman test, as outlined in Figure 14. The test confirmed a significant main effect across the three conversational situations (*p* < 0.05). The post hoc analysis pinpointed significant differences not only between the face-to-face and face-to-video scenario (z = −2.129, *p* < 0.05) but also between face-to-video and face-to-digital human interactions (z = −2.197, *p* < 0.05). When analyzing gaze expression methods in the face-to-video context, significant differences emerged (z = −2.060, *p* < 0.05).

**Figure 15:**
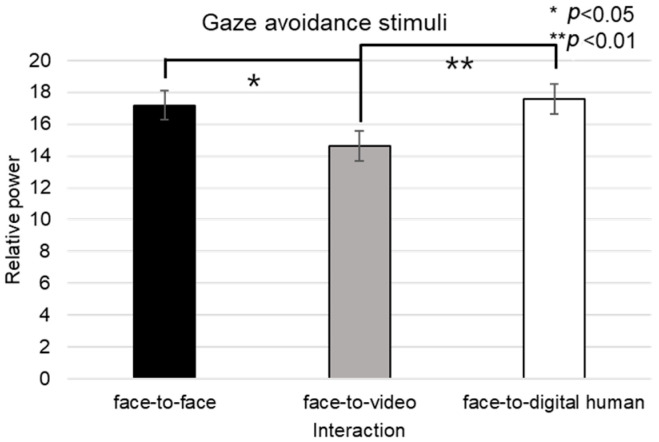
Analysis of brainwave channel T4 in response to negative stimuli.

In the case of the theta-band data from channel T4 under gaze-avoidance stimuli, the normality tests also yielded non-parametric results, leading to the use of the Friedman test for our analysis, as outlined in Figure 15. A significant main effect was observed across the conversational conditions (*p* < 0.05). Post hoc tests showed notable differences between the face-to-face and face-to-video conditions (z = −2.043, *p* < 0.05), as well as between the face-to-video and face-to-digital human conditions (z = −2.641, *p* < 0.01).

### 4.3. Gaze Results

#### 4.3.1. Positive Stimuli

When positive stimuli were presented, we aimed to investigate whether there were differences in the gaze movements of participants.

##### Left Eye

**Figure 16:**
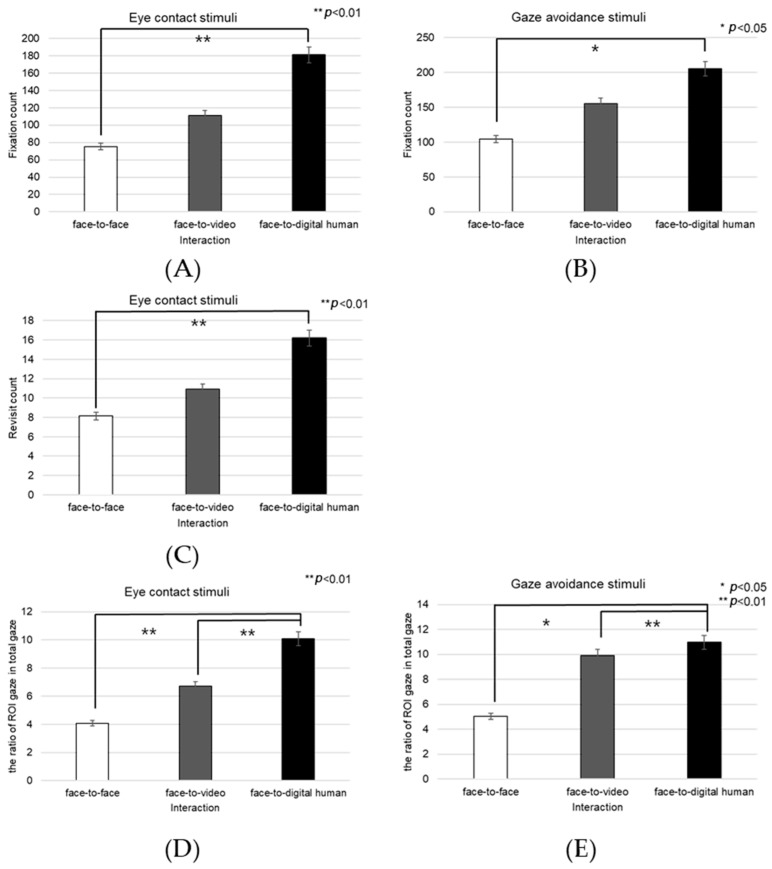
Analysis of gaze to the left eye in response to positive stimuli. (**A**) Fixation count under the eye-contact condition. (**B**) Fixation count under the gaze-avoidance condition. (**C**) Fixation count under the face-to-face condition. (**D**) Revisit counts in eye-contact condition. (**E**) Revisit count under the face-to-video condition.

Analyzing the left eye region, we found a significant effect on the number of fixations in response to positive stimuli across three conversational contexts—face-to-face, face-to-video, and face-to-digital human—when participants engaged in gaze alignment (F(2, 99) = 5.29, *p* < 0.006), as outlined in Figure 16A. Subsequent post hoc analysis highlighted marked differences between the face-to-face and face-to-digital human scenarios (*p* < 0.006).

During instances of gaze avoidance, similar significant effects were noted in the fixation counts within the left eye area across these conversational contexts (F(2, 99) = 4.356, *p* < 0.012), with face-to-face and face-to-digital human comparisons once again showing significant divergence (*p* < 0.012), as outlined in Figure 16B.

In terms of revisits to the left eye area, a significant effect emerged when contrasting the three types of conversational interaction with aligned gaze (F(2, 99) = 6.482, *p* < 0.002). The differences were especially pronounced between face-to-face and face-to-digital human encounters (*p* < 0.002), as outlined in Figure 16C.

Regarding the ratio of fixation on the left eye area versus that on the total area, significant differences were noted when comparing the three conversational situations with aligned gaze (F(2, 99) = 11.087, *p* < 0.018), revealing substantial variances between both face-to-face and face-to-digital human (*p* < 0.018), as well as face-to-video and face-to-digital human interactions (*p* < 0.000), as outlined in Figure 16D.

With gaze avoidance, the fixation ratio within the left eye area also displayed significant changes across conversational contexts (F(2, 99) = 5.719, *p* < 0.032), particularly between face-to-face and face-to-digital human (*p* < 0.032), as well as between face-to-video and face-to-digital human interactions (*p* < 0.008), as outline in Figure 16E.

**Figure 17:**
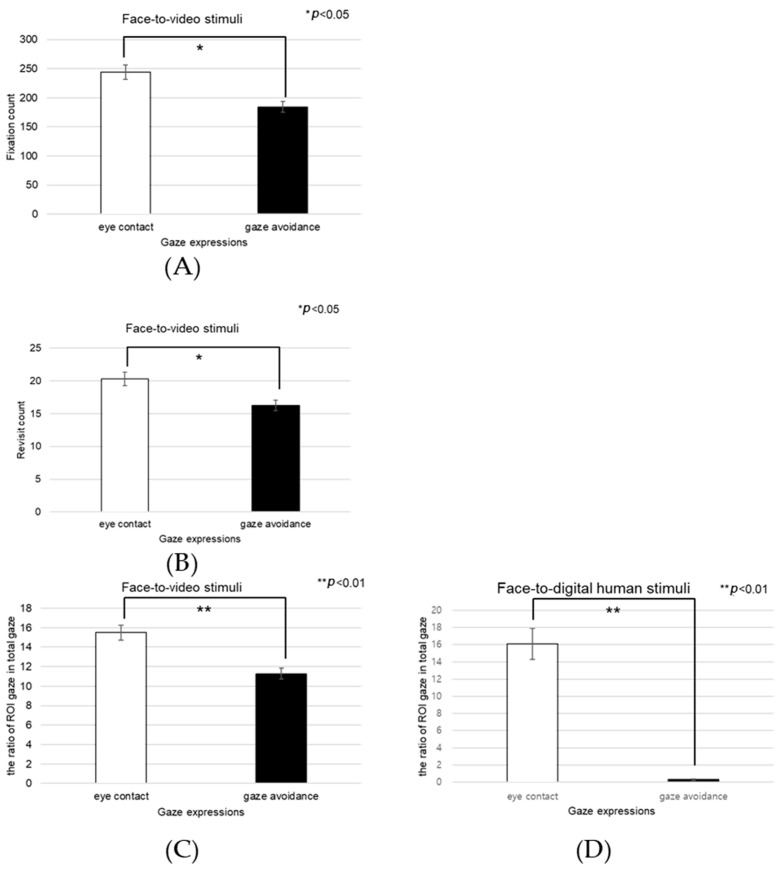
Analysis of gaze to the left eye area in response to positive stimuli. (**A**) Fixation count under the face-to-face condition. (**B**) Revisit count under the face-to-video condition. (**C**) Ratio of ROI under the face-to-video condition. (**D**) Ratio of ROI under the face-to-digital human condition.

When participants viewed face-to-video stimuli, the fixation count within the left eye varied significantly depending on adopted gaze expression (t = −2.743, *p* < 0.01), as outlined in Figure 17A.

Additionally, with face-to-video stimuli, revisits to the left eye were significantly influenced by the method of gaze expression used (t = −3.172, *p* < 0.003), as outlined in Figure 17B.

When examining face-to-video stimuli, significant differences in the fixation ratio within the left eye area were influenced by gaze expression methods (z = −3.257, *p* < 0.001). Similarly, with face-to-digital human stimuli, the fixation ratio within the left eye area significantly varied based on the gaze expression approach (z = −5.069, *p* < 0.001), as outlined in Figure 17C,D.

##### Right Eye

**Figure 18:**
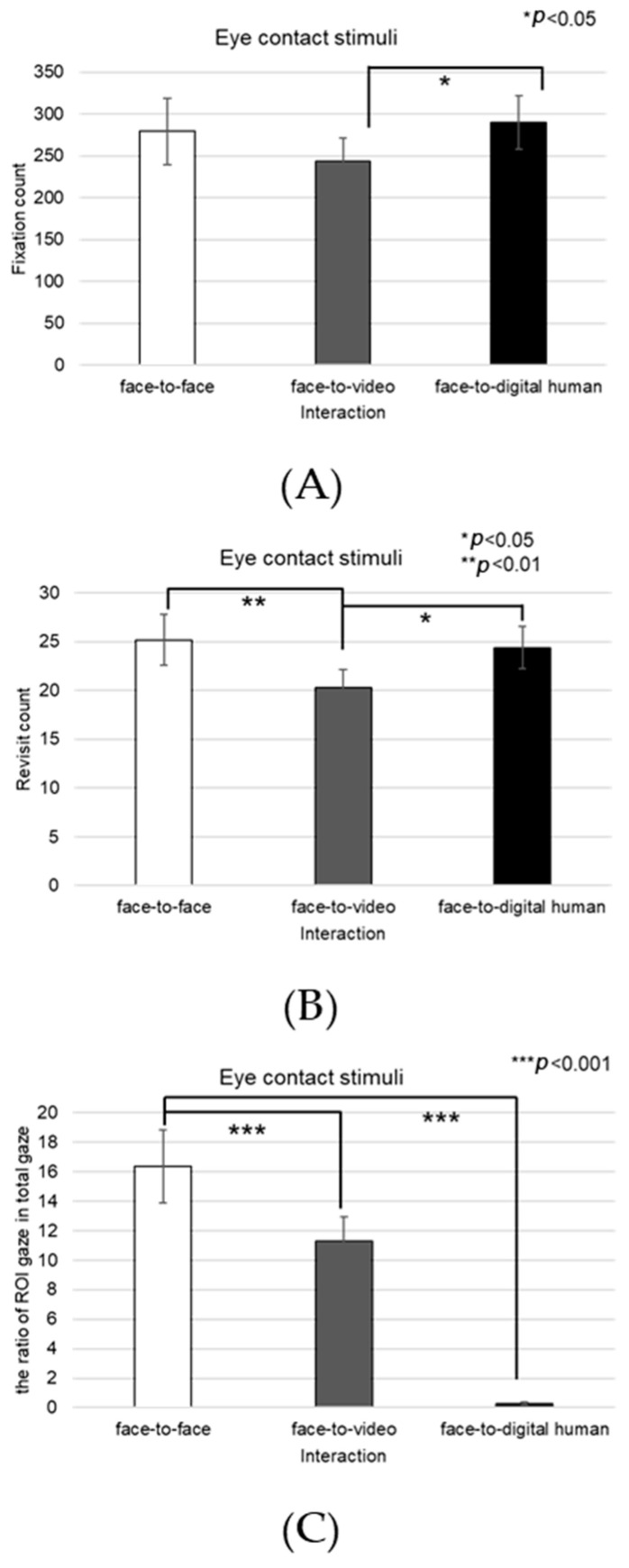
Analysis of gaze to the right eye area in response to positive stimuli. (**A**) Fixation count under the eye-contact condition. (**B**) Revisit count under the eye-contact condition. (**C**) Ratio of ROI under the eye-contact condition.

Fixation frequency on the right eye region exhibited a significant effect across the three conversational situations—face-to-face, face-to-video, and face-to-digital human—when participants demonstrated gaze avoidance (F(2, 99) = 176.01, *p* < 0.017), as outlined in Figure 18A. Post hoc comparisons revealed notable differences specifically between face-to-video and face-to-digital human interactions (*p* < 0.017). Additionally, the revisit count to the right eye area presented a significant effect in relation to the conversational contexts when gaze avoidance was evident (F(2, 99) = 5.410, *p* < 0.009), as outlined in Figure 18B. Here, post hoc analysis distinguished significant contrasts between face-to-face and face-to-video (*p* < 0.009), as well as between face-to-video and face-to-digital human interactions (*p* < 0.032).

The fixation ratio within the right eye area relative to the total observation field showed a significant variance across the conversational settings with expressed gaze avoidance (F(2, 99) = 22.973, *p* < 0.000), as outlined in Figure 18C. Detailed post hoc evaluations pinpointed substantial differences between the face-to-face and face-to-digital human conditions (*p* < 0.000), as well as between the face-to-video and face-to-digital human interactions (*p* < 0.000).

**Figure 19:**
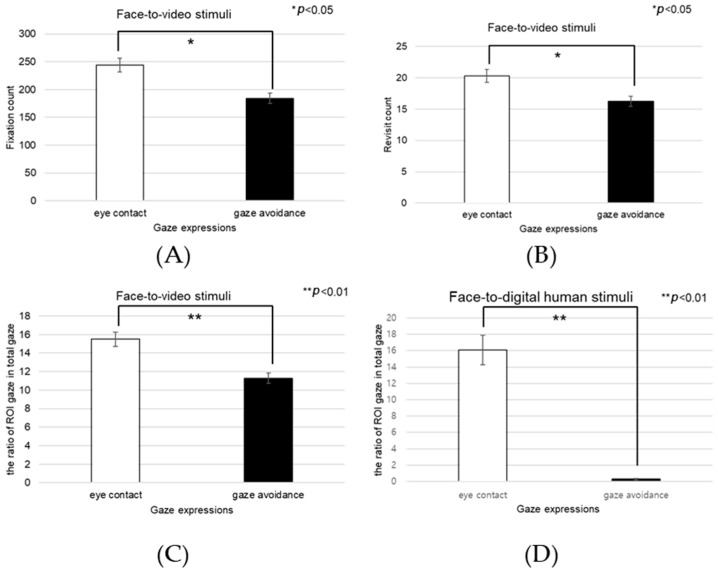
Analysis of gaze to the right eye area in positive stimuli. (**A**) Fixation count under the face-to-video condition. (**B**) Revisit count under the face-to-video condition. (**C**) Ratio of ROI under the face-to-video condition. (**D**) Ratio of ROI under the face-to-digital human condition.

Under conditions involving face-to-video stimuli, the fixation frequency on the right eye was significantly influenced by the method of gaze expression used (t = 2.101, *p* < 0.043), as outlined in Figure 19A.

When subjects were exposed to face-to-video stimuli, the frequency of revisits to the right eye area also differed significantly depending on the gaze expression method employed (t = 2.101, *p* < 0.043), as outlined in Figure 19B.

The fixation ratio within the right eye area also differed significantly in response to face-to-video stimuli and was affected by gaze expression techniques (z = −2.402, *p* < 0.016). A similar significant variation was noted when face-to-digital human stimuli were presented, again depending on the applied gaze expression method (z = −5.035, *p* < 0.001), as outlined in Figure 19C,D.

##### Nose

**Figure 20:**
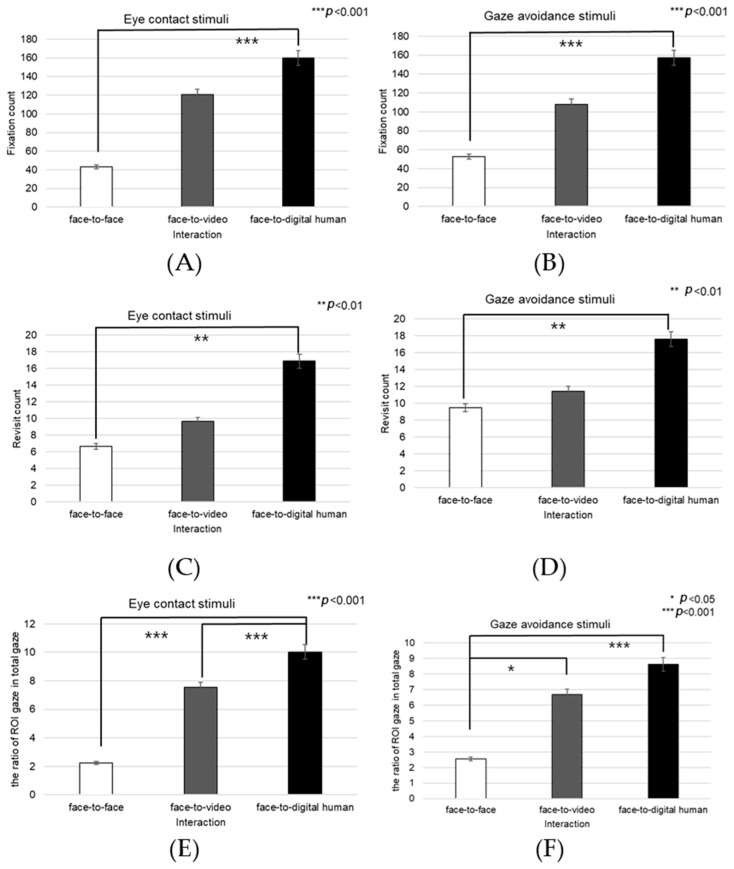
Analysis of gaze to the nose area in response to positive stimuli. (**A**) Fixation count under the eye-contact condition. (**B**) Fixation count under the gaze-avoidance condition. (**C**) Revisit count under the eye-contact condition. (**D**) Revisit count under the gaze-avoidance condition. (**E**) Ratio of ROI under the eye-contact condition. (**F**) Ratio of ROI under the gaze-avoidance condition.

The fixation count on the nose area demonstrated a significant main effect across the three conversational conditions—face-to-face, face-to-video, and face-to-digital human—when aligned gaze was present (F(2, 99) = 9.688, *p* < 0.001), as outlined in Figure 20A. Post hoc testing revealed marked differences particularly between the face-to-face and face-to-digital human scenarios (*p* < 0.001).

With the expression of gaze avoidance, the fixation count in the nose area also showed a significant main effect across these conversational contexts (F(2, 99) = 8.705, *p* < 0.001), as outlined in Figure 20B. Further analysis identified significant disparities between face-to-face and face-to-digital human encounters (*p* < 0.001).

The frequency of revisits to the nose area revealed a significant main effect when evaluating the same three conversational setups with aligned gaze (F(2, 99) = 8.894, *p* < 0.014), as outlined in Figure 20C. Notably, post hoc analysis pointed out significant variations between face-to-video and face-to-digital human interactions (*p* < 0.014).

In instances of gaze avoidance, revisits to the nose area maintained a significant main effect across the conversational contexts (F(2, 99) = 4.868, *p* < 0.011), with significant contrasts identified between face-to-video and face-to-digital human interactions (*p* < 0.011), as outlined in Figure 20D. Furthermore, the ratio of fixations within the nose area relative to the entire observational field presented a significant main effect in the three conversational settings with expressed aligned gaze (F(2, 99) = 10.190, *p* < 0.001). Subsequent post hoc assessments indicated significant distinctions between face-to-face and face-to-video interactions (*p* < 0.001), as well as between face-to-face and face-to-digital human interactions (*p* < 0.001), as outlined in Figure 20E.

Similarly, in response to gaze avoidance, the fixation ratio within the nose area showed a significant main effect when examining the conversational situations (F(2, 99) = 9.202, *p* < 0.018), as outlined in Figure 20F. The post hoc analysis elucidated significant differences between face-to-face and face-to-video interactions (*p* < 0.018), as well as between face-to-face and face-to-digital human interactions (*p* < 0.001).

##### Mouth

**Figure 21:**
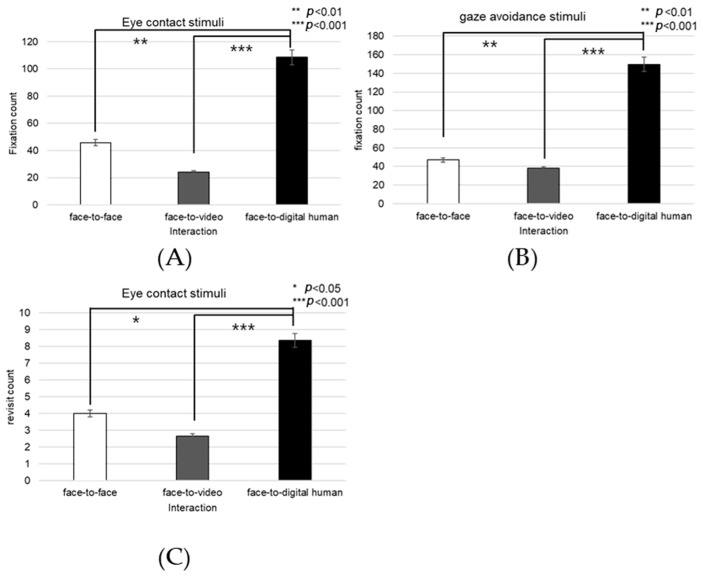
Analysis of gaze to the mouth area in response to positive stimuli. (**A**) Fixation count under the eye-contact condition. (**B**) Fixation count under the gaze-avoidance condition. (**C**) Revisit count under the eye-contact condition.

Significant effects were observed in the fixation count on the mouth area across the three types of conversational interactions—face-to-face, face-to-video, and face-to-digital human—when participants displayed aligned gaze (F(2, 99) = 8.774, *p* < 0.01), as outlined in Figure 21A. Post hoc comparisons revealed substantial differences between face-to-face and face-to-digital human interactions (*p* < 0.01), as well as between face-to-video and face-to-digital human interactions (*p* < 0.001).

In scenarios in which gaze avoidance was evident, a significant effect on the number of fixations in the mouth area was noted across the conversational settings (F(2, 99) = 9.152, *p* < 0.002), as outlined in Figure 21B. The post hoc analysis identified significant disparities between face-to-face and face-to-digital human interactions (*p* < 0.002), as well as between face-to-video and face-to-digital human interactions (*p* < 0.001).

The frequency of revisits to the mouth area also exhibited a significant main effect when contrasting the three conversational situations with aligned gaze (F(2, 99) = 7.470, *p* < 0.017), as outlined in Figure 21C. Upon further analysis, significant differences were discerned between face-to-face and face-to-digital human interactions (*p* < 0.017), as well as between face-to-video and face-to-digital human interactions (*p* < 0.001).

##### Forehead

**Figure 22:**
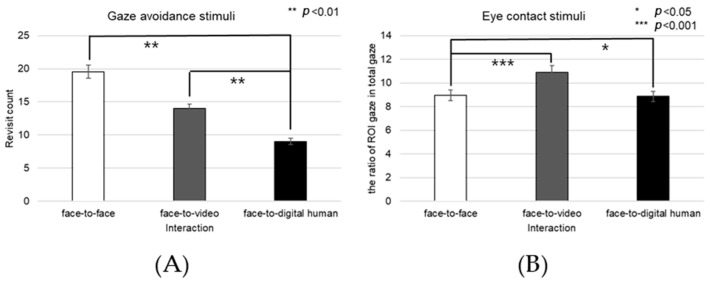
Analysis of gaze to the forehead area in response to positive stimuli. (**A**) Revisit count under the gaze-avoidance condition. (**B**) Ratio of ROI under the eye-contact condition.

When gaze avoidance was displayed, the number of revisits to the forehead area showed a significant main effect across the three conversational conditions—face-to-face, face-to-video, and face-to-digital human (F(2, 99) = 4.778, *p* < 0.017), as outlined in Figure 22A. Post hoc analysis identified notable differences between face-to-face and face-to-digital human interactions (*p* < 0.017), as well as between face-to-video and face-to-digital human interactions (*p* < 0.008). Moreover, the proportion of fixations within the forehead area relative to the entire field displayed a significant main effect in the three conversational scenarios when aligned gaze was present (F(2, 99) = 7.834, *p* < 0.025), as outlined in Figure 22B. Detailed post hoc comparisons revealed substantial discrepancies between face-to-face and face-to-video interactions (*p* < 0.001), as well as between face-to-face and face-to-digital human interactions (*p* < 0.025). The fixation ratio for the forehead area also differed significantly with respect to gaze expression methods when participants were exposed to face-to-face stimuli (z = −2.564, *p* < 0.010).

**Figure 23:**
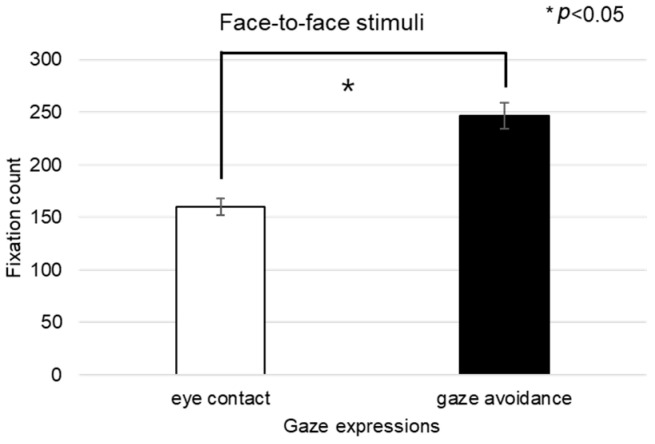
Analysis of fixation count to the forehead under the face-to-face condition in response to positive stimuli.

Fixation counts on the forehead area demonstrated a significant variance associated with different gaze expressions during face-to-face stimuli presentation (t = −2.182, *p* < 0.036), as outlined in Figure 23.

##### Face

**Figure 24:**
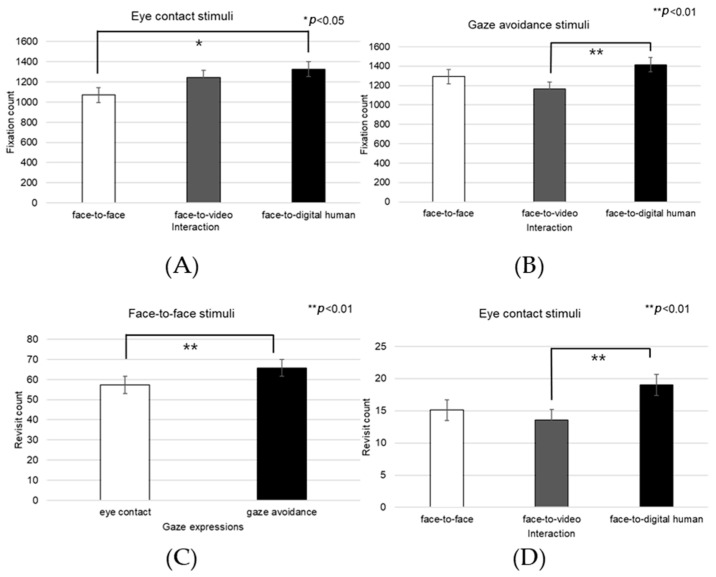
Analysis of gaze to the face area in response to positive stimuli. (**A**) Fixation count under the eye-contact condition. (**B**) Fixation count under the gaze-avoidance condition. (**C**) Revisit count under the face-to-face condition. (**D**) Revisit count under the eye-contact condition.

The fixation count within the facial area was significantly affected across the three conversational scenarios—face-to-face, face-to-video, and face-to-digital human—when participants exhibited aligned gaze (F(2, 99) = 3.216, *p* < 0.043), as outlined in Figure 24A. Post hoc analysis identified a notable discrepancy specifically between face-to-face and face-to-digital human interactions (*p* < 0.043).

With gaze-avoidance behavior, the fixation count within the facial area also showed a significant main effect across the three conversational settings (F(2, 99) = 4.072, *p* < 0.016), as outlined in Figure 24B. Subsequent post hoc examination revealed a significant variation between face-to-video and face-to-digital human interactions (*p* < 0.016).

Additionally, when participants were presented with face-to-face stimuli, the number of facial fixations significantly differed depending on the gaze expression (t = −2.768, *p* < 0.009), as outlined in Figure 24C.

Moreover, the revisit count to the facial area indicated a significant main effect among the three conversational types when aligned gaze was present (F(2, 99) = 5.138, *p* < 0.007), as outlined in Figure 24D. Further analysis through post hoc testing pinpointed a significant contrast between face-to-video and face-to-digital human interactions (*p* < 0.007).

#### 4.3.2. Negative Stimuli

When negative stimuli were presented, we aimed to investigate whether there were differences in the gaze movements of participants.

##### Left Eye

**Figure 25:**
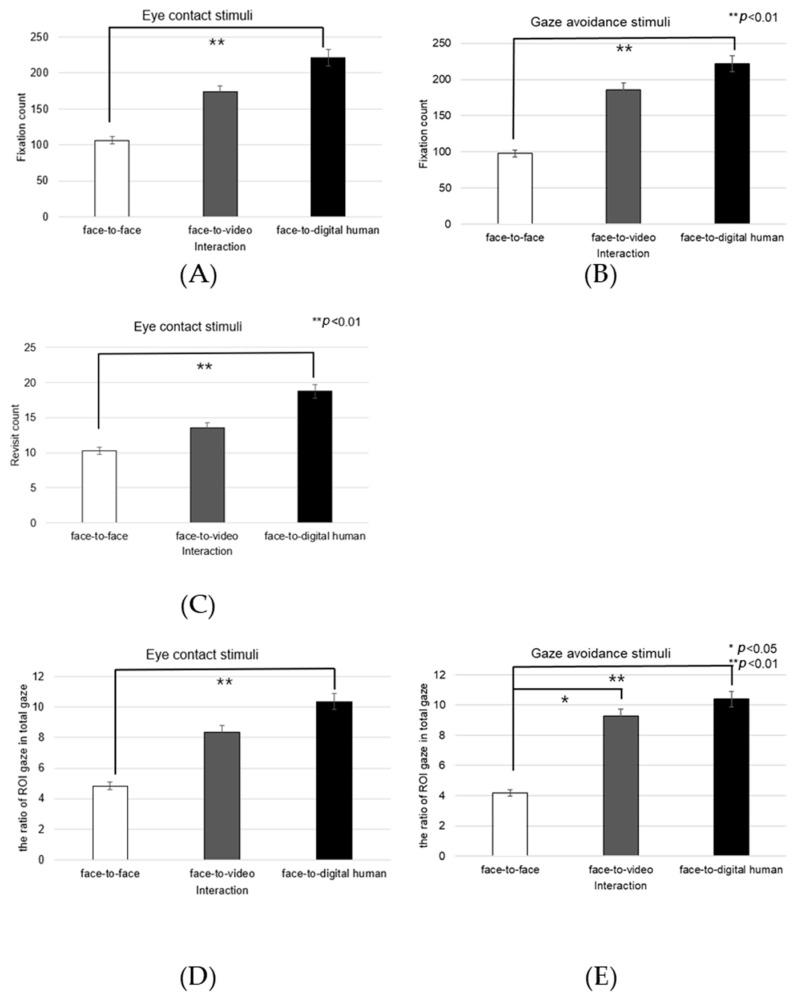
Analysis of gaze to the left eye area in response to negative stimuli. (**A**) Fixation count under the eye-contact condition. (**B**) Fixation count under the gaze-avoidance condition. (**C**) Revisit count under the eye-contact condition. (**D**) Ratio of ROI under the eye-contact condition. (**E**) Ratio of ROI under the gaze-avoidance condition.

In the analysis of the left eye region, significant effects were noted in fixation counts across the three distinct conversational scenarios—face-to-face, face-to-video, and face-to-digital human—when participants engaged in aligned gaze (F(2, 99) = 4.574, *p* < 0.013), as outlined in Figure 25A. Post hoc comparisons highlighted significant variances between face-to-face and face-to-video interactions (*p* < 0.013), as well as between face-to-face and face-to-digital human interactions (*p* < 0.01).

When participants exhibited gaze avoidance, there was a significant main effect on the fixation counts within the left eye area across the conversational contexts (F(2, 99) = 6.381, *p* < 0.012), with notable differences particularly between face-to-face and face-to-digital human interactions (*p* < 0.012), as outlined in Figure 25B.

A significant main effect was also found in the frequency of revisits to the left eye region with aligned gaze (F(2, 99) = 4.687, *p* < 0.009), as outlined in Figure 25C. Further analysis via post hoc testing indicated significant distinctions specifically between face-to-face and face-to-digital human interactions (*p* < 0.009).

The fixation proportion within the left eye area relative to the overall observed area displayed a significant main effect under conditions of aligned gaze (F(2, 99) = 5.027, *p* < 0.007), as outlined in Figure 25D. Subsequent post hoc examination identified significant differences between face-to-face and face-to-digital human scenarios (*p* < 0.007).

Furthermore, with the expression of gaze avoidance, the fixation proportion within the left eye area relative to the entire observed area showed a significant main effect across the conversational settings (F(2, 99) = 6.381, *p* < 0.003), as outlined in Figure 25E. The post hoc analysis revealed significant contrasts between face-to-face and face-to-video interactions (*p* < 0.012), as well as between face-to-face and face-to-digital human interactions (*p* < 0.003).

##### Right Eye

**Figure 26:**
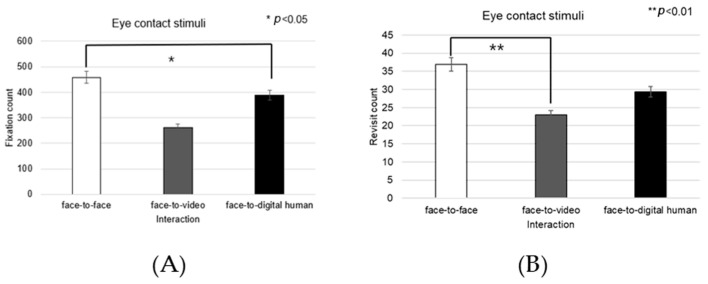
Analysis of gaze to the right eye area in response to negative stimuli. (**A**) Fixation under the eye-contact condition. (**B**) Revisit count under the eye-contact condition.

A significant effect was found in the number of fixations on the right eye area across the three conversational conditions—face-to-face, face-to-video, and face-to-digital human—when participants displayed gaze avoidance (F(2, 99) = 3.807, *p* < 0.023), as outlined in Figure 26A. Post hoc tests identified a significant distinction between the face-to-video and face-to-digital human scenarios (*p* < 0.023).

Additionally, the revisit frequency to the right eye area was significantly affected in the context of gaze avoidance during the three types of conversational interaction (F(2, 99) = 5.485, *p* < 0.004), as outlined in Figure 26B. The post hoc analysis pinpointed a significant difference between the face-to-face and face-to-video settings (*p* < 0.004).

##### Nose

**Figure 27:**
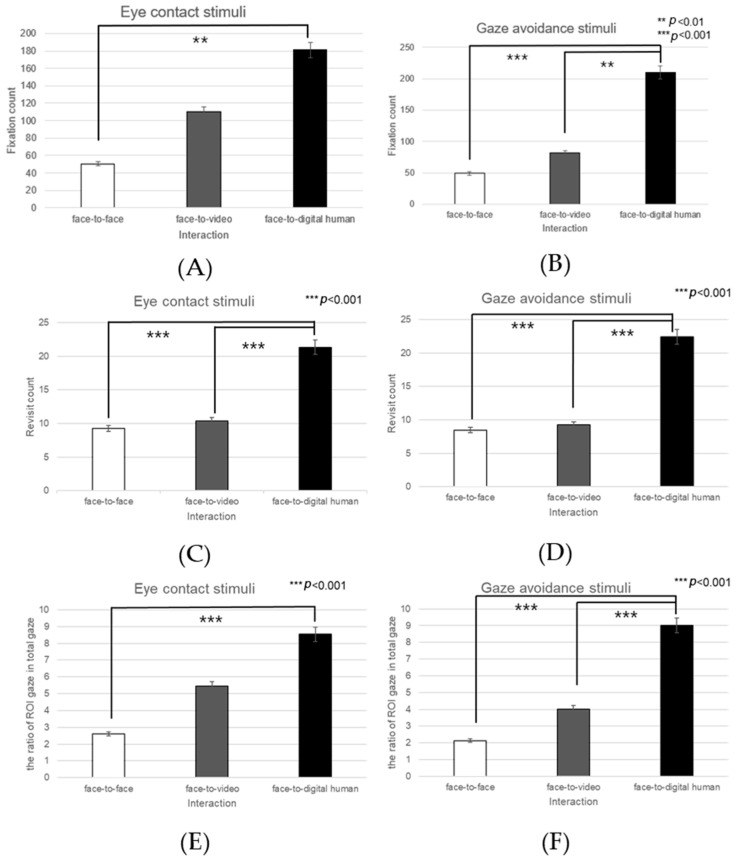
Analysis of gaze to the nose area in response to negative stimuli. (**A**) Fixation count under the eye-contact condition. (**B**) Fixation count under the gaze-avoidance condition. (**C**) Revisit count under the eye-contact condition. (**D**) Revisit count under the gaze-avoidance condition. (**E**) Ratio of ROI under the eye-contact condition. (**F**) Ratio of ROI under the gaze-avoidance condition.

Significant effects were noted in the number of fixations on the nose area under the three conversational conditions—face-to-face, face-to-video, and face-to-digital human—when aligned gaze was exhibited (F(2, 99) = 4.576, *p* < 0.010), as outlined in Figure 27A. Post hoc analysis identified significant variations between face-to-face and face-to-digital human encounters (*p* < 0.010).

With gaze avoidance, the fixation count on the nose area also showed a significant effect across the conversational scenarios (F(2, 99) = 5.357, *p* < 0.006), as outlined in Figure 27B. Subsequent post hoc analysis highlighted a significant disparity between face-to-face and face-to-digital human interactions (*p* < 0.006).

Regarding revisits to the nose area, a significant main effect was observed in the presence of gaze alignment across the conversational contexts (F(2, 99) = 11.707, *p* < 0.001), with a notable difference between face-to-video and face-to-digital human (*p* < 0.001), as outlined in Figure 27C.

When gaze avoidance was demonstrated, revisits to the nose area were significantly affected across the three types of conversational interaction (F(2, 99) = 13.731, *p* <0.001), as outlined in Figure 27D. The post hoc analysis confirmed significant differences between face-to-face and face-to-digital human interactions (*p* < 0.001), as well as between face-to-video and face-to-digital human interactions (*p* < 0.001).

The fixation proportion within the nose area relative to the total observed area also presented a significant main effect with aligned gaze in the three conversational settings (F(2, 99) = 7.681, *p* < 0.001), as outlined in Figure 27E. Upon further analysis, significant contrasts were evident between face-to-face and face-to-digital human interactions (*p* < 0.001).

In response to the expression of gaze avoidance, this fixation proportion within the nose area showed a significant effect in the conversational comparisons (F(2, 99) = 14.749, *p* < 0.001), as outlined in Figure 27F. Post hoc analysis demonstrated significant differences between face-to-face and face-to-digital human interactions (*p* < 0.001).

##### Mouth

**Figure 28:**
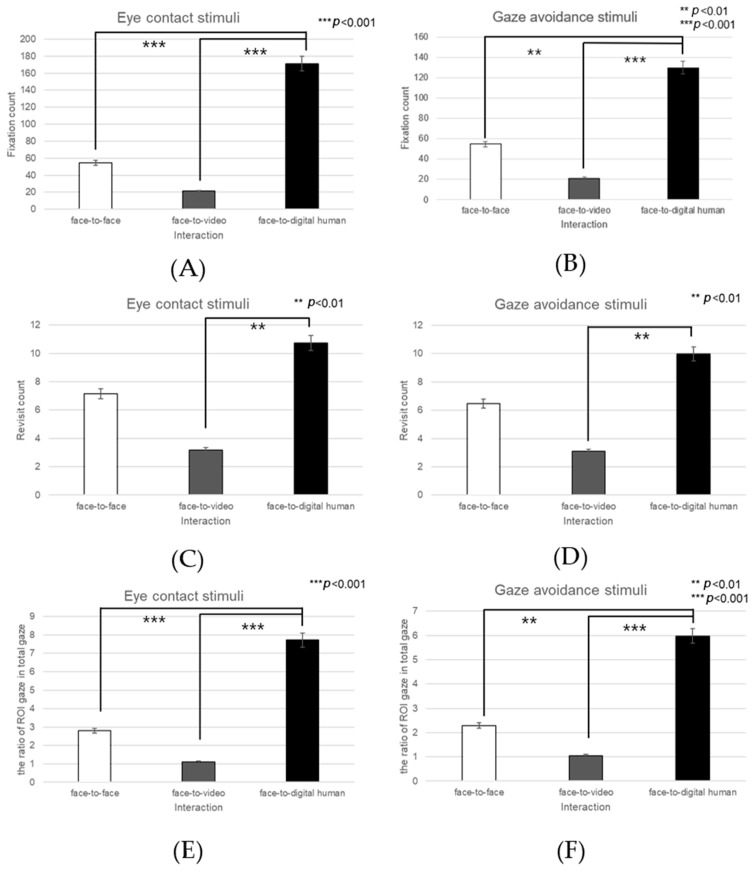
Analysis of gaze to the mouth area in response to negative stimuli. (**A**) Fixation count under the eye-contact condition. (**B**) Fixation count under the gaze-avoidance condition. (**C**) Revisit count under the eye-contact condition. (**D**) Revisit count under the gaze-avoidance condition. (**E**) Ratio of ROI under the eye-contact condition. (**F**) Ratio of ROI under the gaze-avoidance condition.

During gaze alignment expression, a significant main effect was observed in the fixation counts within the mouth area across three conversational contexts—face-to-face, face-to-video, and face-to-digital human (F(2, 99) = 16.945, *p* < 0.001), as outlined in Figure 28A. Post hoc analysis identified pronounced differences between face-to-face and face-to-digital human interactions (*p* < 0.001), as well as between face-to-video and face-to-digital human interactions (*p* < 0.001).

With the expression of gaze avoidance, the fixation counts in the mouth area also demonstrated a significant main effect across these conversational scenarios (F(2, 99) = 10.117, *p* < 0.0029), as outlined in Figure 28B. The post hoc analysis revealed substantial differences between face-to-face and face-to-digital human interactions (*p* < 0.0029), as well as between face-to-video and face-to-digital human interactions (*p* < 0.001).

In terms of revisits to the mouth area during gaze alignment, a significant effect was noted when comparing the conversational types (F(2, 99) = 6.138, *p* < 0.002), as outlined in Figure 28C. Upon further examination, significant distinctions were found between face-to-video and face-to-digital human interactions (*p* < 0.002).

Similarly, when gaze avoidance was expressed, the revisit frequency to the mouth area showed a significant effect across the conversational conditions (F(2, 99) = 5.467, *p* < 0.004), as outlined in Figure 28D. Post hoc analysis indicated a significant difference specifically between face-to-video and face-to-digital human interactions (*p* < 0.004).

Regarding the proportion of total-area fixations on the mouth area with aligned gaze, a significant main effect emerged in the conversational comparisons (F(2, 99) = 15.174, *p* < 0.001), as outlined in Figure 28E. The post hoc analysis brought to light significant discrepancies between face-to-face and face-to-digital human interactions (*p* < 0.001).

When gaze avoidance was the focus, the proportional fixation within the mouth area exhibited a significant main effect across the three conversational scenarios (F(2, 99) = 10.984, *p* < 0.003), as outlined in Figure 28F. Post hoc assessments showed significant contrasts between face-to-face and face-to-digital human interactions (*p* < 0.003), as well as between face-to-video and face-to-digital human interactions (*p* < 0.001).

##### Forehead

**Figure 29:**
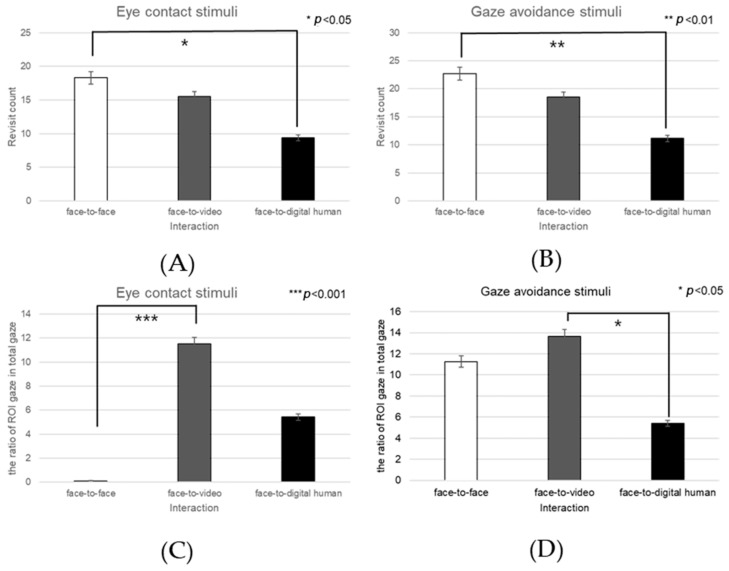
Analysis of gaze to the forehead area in response to negative stimuli. (**A**) Revisit count under the eye-contact condition. (**B**) Revisit count under the gaze-avoidance condition. (**C**) Ratio of ROI under the eye-contact condition. (**D**) Ratio of ROI under the gaze-avoidance condition.

The frequency of revisits to the forehead area during the expression of aligned gaze exhibited a significant main effect across the face-to-face, face-to-video, and face-to-digital human conversational contexts (F(2, 99) = 3.572, *p* < 0.031), as outlined in Figure 29A. Post hoc analysis detected a significant variance between face-to-face and face-to-digital human encounters (*p* < 0.031)

Under conditions of gaze avoidance within the forehead area, a significant main effect was noted in comparisons among the three conversational types (F(2, 99) = 4.933, *p* < 0.008), with significant differences identified between face-to-face and face-to-digital human interactions (*p* < 0.008), as outlined in Figure 29B.

When aligned gaze was expressed, the proportion of fixations focused on the forehead area relative to the total area demonstrated a significant main effect in the conversational comparisons (F(2, 99) = 6.981, *p* < 0.001), as outlined in Figure 29C. Upon further analysis, significant discrepancies were revealed between face-to-face and face-to-video interactions (*p* < 0.001).

With gaze avoidance in the forehead region, a significant main effect emerged across the conversational scenarios (F(2, 99) = 3.531, *p* < 0.034), as outlined in Figure 29D. Post hoc analysis indicated a notable difference specifically between face-to-video and face-to-digital human interactions (*p* < 0.034).

**Figure 30:**
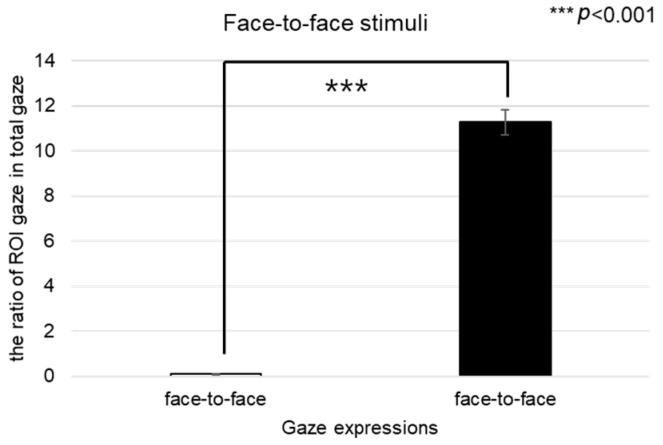
Analysis of the ratio of ROI to the forehead under the face-to-face condition in response to negative stimuli.

Additionally, a significant differentiation based on gaze expression methods was found in the forehead area when participants were presented with face-to-face stimuli (t = −5.259, *p* < 0.000), as outlined in Figure 30.

### 4.4. Analysis of the Hypothesis

The aim of this study was to understand if engagement varies according to conversation conditions and gaze expressions. Hypothesis H1 was posited to validate the differences in engagement across three conversational situations. Only H1-1 was supported under the condition in which eye contact was established, with a difference in engagement observed between face-to-face and face-to-digital human conversations under both emotional conditions (positive and negative). Table 4 depicts the characteristics of each conversational condition to highlight the degree of engagement.

Among the characteristics, the face-to-face and non-face-to-face situations marked in gray did not show a statistically significant difference, but the face-to-face situation was associated with higher engagement than the non-face-to-face situation. This suggests that while there are differences in media between face-to-face and non-face-to-face situations, no statistically significant difference occurred because the conversational partner was a person in both cases.

In terms of the characteristics of the conversational target, the non-face-to-face and digital-human situations marked in gray did not show a statistically significant difference, but the non-face-to-face situation was associated with higher engagement than the digital-human situation. This suggests that there is a difference in engagement depending on the conversational target in non-face-to-face and digital-human situations, but because the same media provided the stimulus, the difference in engagement was not statistically significant.

Hypothesis H2 was intended to test for differences in engagement according to gaze expressions. When positive emotional stimuli were presented, differences in engagement occurred in the three conversational situations based on eye contact and gaze avoidance. Consequently, hypotheses H2-1, H2-2, and H2-3 were confirmed only in the context of positive emotions.

Hypothesis H3 tested for differences in engagement between positive and negative emotions. H3 found support solely under conditions of eye-contact expressions across all three conversational contexts.

In summary, the results indicate that to enhance user engagement, positive emotional stimuli should be considered over negative stimuli. Additionally, eye-contact expression should be used to induce increased engagement.

## 5. Discussion and Conclusions

Results of subjective evaluations based on gaze expressions revealed differences in engagement across the three conversation conditions, along with physiological responses to gaze engagement (i.e., eye contact) and avoidance. However, in terms of subjective evaluations, differences were observed only between face-to-face and digital-human conversations. In contrast, physiological responses to gaze showed inconsistencies, differing from subjective evaluations under conditions like face-to-face versus face-to-video and face-to-video versus digital-human conversations.

One can tell by a person’s eye movements where they are looking, what they are seeing, and what they are choosing [45]. Gaze variables, in particular, can be used to acquire information of interest or attention, and one tends to spend more time looking at things of interest [46,47]. The discrepancy between the subjective evaluation results and the physiological gaze reactions may be interpreted as the participants finding the digital-human stimuli a novel and unfamiliar experience, piquing their interest more than the relatively familiar face-to-face and face-to-video conversations, leading to higher gaze metrics for digital-human stimuli.

According to the gaze results of this study, in digital-human conversation, when eye contact was met, the number of gaze fixations, the ratio of fixations within the area of interest to the entire area, and the number of revisits—gaze variables associated with interaction, passive participation, and high concentration—were higher compared to the face-to-face and face-to-video conditions. Likewise, in digital-human conversations, when gaze avoidance was expressed, these gaze variables were found to be higher compared to face-to-face and face-to-video stimuli. The valid gaze variables identified by these results—the number of gaze fixations, the ratio of the area of interest within the total area, and the number of revisits—align with the outcomes of previous social–emotional research [48]. This suggests that gaze variables can be utilized to measure social emotions using digital-human stimuli.

Under digital-human conversation conditions, the number of gaze fixations and revisits, as well as the proportion of the area of interest within the entire area, were found to be higher in the mouth area compared to face-to-face and face-to-video conversations. People read the mouth and facial movements of others, including lip movements, to understand spoken language [49,50]. Although lip-sync animations for the digital-human conversation stimuli were created based on the experimenter’s mouth movement habits using Unity, due to technical limitations, the digital human’s lip movements were not expressed as naturally as those of real humans. Therefore, it can be interpreted that under digital-human conditions, it was more difficult for users to gather information from the lip movements, leading to an increase in the number of gaze fixations, the proportion of the area of interest within the entire area, and revisits in the mouth area compared to face-to-face and non-face-to-face situations.

**Figure 31:**
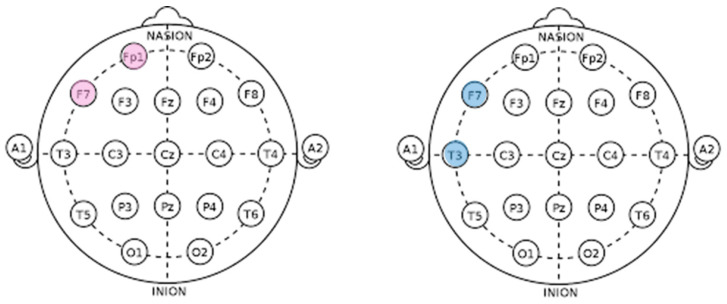
EEG results in response to gaze expression.

The EEG results of this study indicated that in response to positive stimuli, channels FP1 and F7 were activated, whereas in response to negative stimuli, channels F7, T3, and T4 were activated. Notably, results with respect to gaze alignment and avoidance showed activation in the left hemisphere’s FP1 and F7 channels for positive stimuli and in the left hemisphere’s F7 and T3 channels for negative stimuli (see Figure 31).

When comparing areas of interest such as the left eye, right eye, nose, mouth, forehead, and the entire face, it was observed that the right eye area was fixated upon more than other areas. Eye movement is associated with cognitive characteristics, and when the eyes move to the left, the right brain is activated, whereas when moving to the right, the left brain is activated [51]. The EEG results showed activation in the left brain, which can be interpreted as a result of fixating more on the right eye area compared to other areas of interest, such as the left eye, nose, mouth, and forehead.

In summary, to enhance engagement in digital-human conversation, the following steps should be considered:

1. Users should be provided with a positive experience. In this study, storytelling stimuli were used to evoke positive experiences, employing themes that are easily encountered in daily life, such as successful achievement; that is, positive storytelling stimuli may induce more user engagement than negative storytelling stimuli.

2. It should feel natural for users to make eye contact with the digital human. Using digital-human stimuli to induce eye contact with users is a challenging task that requires careful consideration. Previous research related to gaze alignment and avoidance in non-face-to-face situations [37] lacks guidelines for inducing gaze alignment for experimentation. However, in this study, we referred to previous work in which researchers manipulated eye contact between robots and humans [36] to achieve more accurate gaze expression effects; that is, participants were guided to adjust a height-adjustable desk until they felt they had achieved eye contact under the digital-human conditions. In addition to evaluating whether they felt eye contact through subjective evaluation, gaze analysis confirmed that participants fixated on the right eye area for longer periods or revisited it several times through gaze analysis.

To the best of our knowledge, this research is the first to verify the effects of engagement by considering levels of face-to-face interaction, non-face-to-face interaction (i.e., face-to-video), and digital-human conversation conditions with ecological validity. Table 5 outlines our contributions compared to the previous literature.

We made the digital human’s appearance to look as real as possible and produced a digital human with the gaze-avoidance habits of the experimenter. However, there were parts where the skeletal structure, lip shape, and eye shape could not be perfectly replicated due to technical limitations. Also, when creating the digital human’s lip sync, it was intended to reflect the experimenter’s lip movements as closely as possible, but there were unnatural aspects in the graphic movements, making the lip-sync appear awkward. Due to technical limitations, the experimenter’s habit-reflected lip-sync could not be applied to the digital human in real time, resulting in the assumption of one-way communication rather than interactive dialogue. Future research should overcome these technical limitations to enable interactive communication and test the engagement effects with more natural-looking digital humans without the need for preadjustment of eye contact.

In this study, the robustness and interpretability of ANOVA were crucial, allowing for clear conclusions about our hypotheses, which are essential with respect to the contribution we aim to make to the existing body of knowledge. However, we acknowledge the possibility of using more recent machine learning methods. In future research, where the objectives might be more aligned with predictive analysis, or when exploring data-driven hypotheses, we see great value in employing these advanced methods.

The results of this study allowed for the verification of effects on engagement depending on the conversational situation and gaze expression through subjective evaluations and analysis of biometric signals (i.e., eye movement and EEG). In short, using positive stimuli can enhance user engagement with digital humans, as making digital humans initiate eye contact tends to foster greater engagement.

## Figures and Tables

**Table 1:** Positive sentence stimuli used in the experiment.

Number	Positive Sentence
1	I applied to three graduate schools for this semester, but I really messed up the interview for the school I wanted to go to the most. I thought for sure I wouldn’t get accepted, but just to make sure, I checked, and it said congratulations on your acceptance.
2	Last semester, I had a psychology class among my major courses, and the professor was known for setting difficult exam. Instead of a midterm exam, we had weekly quizzes, and the final exam covered the entire course, so I was worried. But I ended up getting the highest score on the final exam and received an A+.
3	My cousin’s liver is not in good condition, and it seems that the situation has worsened. The doctor said that a liver transplant is necessary. However, my cousin’s father has severe fatty liver, so the liver transplant cannot be performed. But he did diet and they have decided to proceed with the surgery. I hope they recover quickly so we can go out and have fun together.
4	Yesterday, a fire broke out in the mountains behind my house, so we had to evacuate. I was worried about the cats that I occasionally fed, wondering if they managed to evacuate safely. I started searching for the cats around the area, and fortunately, during the firefighting, the firefighters found the cats and moved them to a safe place.
5	I decided to take a leave of absence for the next semester to prepare for an internship. I diligently prepared for an internship at a company I really want to work for, but I didn’t share this with people around me because there was a possibility of not getting accepted. However, yesterday, I received a call expressing their interest in interviewing me. I just need to pass the interview to secure the internship.
6	My dog often goes outside, so I always make sure to keep a close eye on the house door. However, while I briefly opened the door to receive something, my dog managed to slip out of the house. After some time had passed, I realized that my dog had gone outside, so I searched around the neighborhood, but couldn’t find them. I returned home to make a report, but my dog was waiting for me in front of the house.

**Table 2:** Negative sentence stimuli used in the experiment.

Number	Negative Sentence
1	Yesterday, my dog suddenly passed away. It was strange that my dog died so suddenly, so I examined the vomit and found something resembling blue rodenticide. Since I don’t use rodenticides, I checked the CCTV in my yard. It revealed that a neighbor, whom I had a dispute with regarding my dog a few days ago, came and secretly scattered the rodenticide before leaving. They deliberately caused the death of my dog.
2	Yesterday, my brother’s child went on a picnic with her kindergarten. However, the bus carrying the kindergarten children was involved in an accident with a dump truck driven by a drowsy driver. My brother’s child was on that bus, and all the children and teachers on the bus lost their lives on the spot.
3	My friend recently had a newborn baby. However, on a day when my friend was working overtime, their house was invaded by a burglar. The burglar held the baby hostage and threatened my friend’s wife, demanding money. Despite my friend’s wife giving the burglar money, the burglar became demand more and in a fit of rage, the burglar ended up killing the baby.
4	My boyfriend commutes on the highway, and yesterday there seemed to be a lot of traffic, so he had to brake suddenly. However, a trailing truck failed to brake in time and collided with my boyfriend’s car at high speed. Sadly, my boyfriend passed away at the scene of the accident.
5	Among the dogs I have, there is one that tends to go outside the house, so I always make sure to keep an eye on the doors. However, during a momentary distraction, the dog managed to slip out of the house, and I immediately followed to search for it. Unfortunately, I couldn’t find the dog, so I reported it as missing on an app, but I haven’t received any reports yet.
6	Yesterday, someone secretly started a fire by burning trash in the mountains behind my house. Due to the dry air lately, the fire quickly spread, and I had to evacuate. While evacuating, I couldn’t help but worry about the stray cats that I occasionally took care of. After the fire was extinguished, I went searching for the cats, but I noticed a crowd gathering in one place. When I approached, I discovered that those cats had perished in the fire.

**Table 3:** Measurement items used in the subject evaluation.

Measurement Item	M	SD	Factor			
			1	2	3	4
Engagement	4.258	0.232				
I was excessively engaged when I heard this story.	5.492	1.378	0.871			
I was really drawn into the story.	4.808	1.557	0.864			
Time seemed to slip away when I became engrossed in the story.	4.333	1.697	0.842			
I felt as if I were a character within the story.	3.000	1.680	0.828			
I lost self-awareness while immersed in listening to the story.	3.592	1.780	0.824			
I experienced a sense of participation in the story.	5.033	1.582	0.819			
I felt as if the story was really happening to me.	3.517	1.829	0.808			
While listening to the story, I felt as if I were part of the narrative itself.	2.249	1.640	0.805			
As I listened to the story, I became fully engrossed.	6.058	1.079	0.695			
Identification empathy	4.100	0.112				
I felt that the situation in the story has similarities to the situations I have experienced so far (or may experience in the future).	3.875	1.790		0.918		
I felt that a similar situation could happen to me as well.	4.992	1.606		0.874		
I felt that the emotions experienced by the characters in the story are similar to the emotions I have experienced so far (or may experience in the future).	3.433	1.809		0.837		
Cognitive empathy	3.753	0.131				
I understood the context of the story.	3.433	1.804			0.878	
I understood how the characters in the story feel.	4.508	1.556			0.731	
I found myself mirroring the emotions of the speaker.	3.317	1.754			0.579	
Narrative-induced emotion	3.542	0.200				
Listening to the story was fun.	2.700	1.908				0.947
I was moved by the story.	4.383	1.625				0.947
Eigenvalue			6.637	2.688	2.266	1.922
Cronbach’s α			0.956	0.905	0.787	0.912

**Table 4:** Characteristics of conversation conditions. Gray shading (background) indicates that the condition did show differences in engagement but did not show statistically significant differences.

Conversation Condition	Characteristics	
	Medium	Conversation Dyad
Face-to-face	Non-digital	Human
Face-to-video	Digital	Human
Face-to-digital human	Digital	Digital human

**Table 5:** The contribution of this study compared to the previous literature.

Literature	Similarities	Differences (Our Contributions)
[52]	Comparison of interaction between humans and non-human entities (i.e., robots).	Our research focuses explicitly on eye avoidance and eye contact within the context of conversations, whereas the cited study explores nonverbal interactions under different circumstances. Additionally, in contrast to the cited study, which employs a Nao robot, our approach utilizes a virtual human designed to resemble a real human.
[53]	Assesses human engagement with robots based on human gaze patterns.	Our study differs in that it focuses on more natural conversational interactions as opposed to the question-and-answer dialogue format used in the cited study. Additionally, we conducted a comparative analysis between human-to-human interactions and human-to-virtual human interactions—an aspect not explored in the cited study.
[29]	Evaluate human engagement with a digital human during conversational interactions.	While the cited study assessed interactions with a digital human, it did not include comparisons with human (participant)-to-human conditions—an aspect that our research specifically addresses.

## Data Availability

Data are contained within the article.
